# Multiplexed in vivo homology-directed repair and tumor barcoding enables parallel quantification of Kras variant oncogenicity

**DOI:** 10.1038/s41467-017-01519-y

**Published:** 2017-12-12

**Authors:** Ian P. Winters, Shin-Heng Chiou, Nicole K. Paulk, Christopher D. McFarland, Pranav V. Lalgudi, Rosanna K. Ma, Leszek Lisowski, Andrew J. Connolly, Dmitri A. Petrov, Mark A. Kay, Monte M. Winslow

**Affiliations:** 1 Department of Genetics, Stanford University School of Medicine, Stanford, CA 94305 USA; 2 Department of Pediatrics, Stanford University School of Medicine, Stanford, CA 94305 USA; 3 Department of Biology, Stanford University, Stanford, CA 94305 USA; 4 Translational Vectorology Group, Children’s Medical Research Institute, Westmead, NSW 2145 Australia; 5 Military Institute of Hygiene and Epidemiology, Puławy, 24-100 Poland; 6 Department of Pathology, Stanford University School of Medicine, Stanford, CA 94305 USA; 7 Cancer Biology Program, Stanford University School of Medicine, Stanford, CA 94305 USA; 8 Stanford Cancer Institute, Stanford University School of Medicine, Stanford, CA 94305 USA

## Abstract

Large-scale genomic analyses of human cancers have cataloged somatic point mutations thought to initiate tumor development and sustain cancer growth. However, determining the functional significance of specific alterations remains a major bottleneck in our understanding of the genetic determinants of cancer. Here, we present a platform that integrates multiplexed AAV/Cas9-mediated homology-directed repair (HDR) with DNA barcoding and high-throughput sequencing to simultaneously investigate multiple genomic alterations in de novo cancers in mice. Using this approach, we introduce a barcoded library of non-synonymous mutations into hotspot codons 12 and 13 of *Kras* in adult somatic cells to initiate tumors in the lung, pancreas, and muscle. High-throughput sequencing of barcoded *Kras* ^*HDR*^ alleles from bulk lung and pancreas reveals surprising diversity in Kras variant oncogenicity. Rapid, cost-effective, and quantitative approaches to simultaneously investigate the function of precise genomic alterations in vivo will help uncover novel biological and clinically actionable insights into carcinogenesis.

## Abstract (web summary)

Genome editing technologies enable the rapid interrogation of genetic alterations. Here, the authors present a CRISPR/Cas9-based platform to simultaneously investigate multiple activating point mutations in de novo cancers in mice; and generate panels of Kras-variants in different tissues to induce cancer.

## Introduction

Although somatic mutations in oncogenes typically cluster within a few specific residues, the resulting amino acid changes can produce diverse oncogenic variants that have dramatically different biochemical properties and which correlate with distinct clinical outcomes^1,2^. The functional consequences of specific oncogenic alterations have been investigated in cell lines and in genetically engineered mouse models. Overexpression of putative oncogenic variants in cell lines can uncover some aspects of their function, but the interpretion of these studies is complicated by the non-physiologic expression of variants, presence of cell line-specific background alterations, and lack of a native in vivo environment^3^. Conversely, tumors in genetically engineered mice are driven by defined mutations expressed at physiological levels and develop within their natural context. Tumors initiated in these autochthonous mouse models also recapitulate the gene expression programs and histopathological progression of human cancers, including the development of invasive and metastatic disease^4^. However, genetically engineered mouse models are greatly limited by the time and cost associated with their development and use^4^.

*KRAS* is the most frequently mutated oncogene in human cancer^5^. Given the immense importance of oncogenic RAS proteins in human cancer, and the renewed hope for therapeutics targeting this family of proteins, there has been a resurgence of interest in understanding the functional properties of diverse oncogenic variants of RAS proteins^6^. *KRAS* commonly harbors non-synonymous point mutations in codon 12 or 13 that result in diverse amino acid substitutions. Interestingly, certain point mutations in *KRAS* codon 12 or 13 are identified much more frequently than others, which is thought to be a product of non-uniform mutation rates as well as biological differences between distinct oncogenic KRAS variants^1,7,8^.

Although conventional genetically engineered mice have been used to model oncogenic KRAS-driven cancers, very few Kras variants have been studied in autochthonous mouse models. For instance, oncogenic KRAS-driven lung cancer has been almost exclusively modeled using knock-in alleles in which Cre-mediated or spontaneous recombination leads to Kras^G12D^ expression^9,10^. However, KRAS^G12D^ represents <20% of KRAS mutations in human lung adenocarcinoma^5,11^. Nonetheless, data from Kras^G12D^-based mouse models are often extrapolated to make claims about “oncogenic KRAS” in general, while pre-clinical studies performed in mice with Kras^G12D^-driven tumors may not predict the response of all oncogenic KRAS-driven human tumors. Our limited understanding of the in vivo oncogenicities of even the most common variants of KRAS underscores the fundamental and urgent need for rapid, quantitative, and precise in vivo methods to model the diverse genetic alterations that occur in human cancers.

CRISPR/Cas9-mediated somatic genome editing has recently been used to rapidly investigate the function of tumor suppressor genes in several autochthonous mouse models of cancer^4,12^. Although the CRISPR/Cas9 system can be used to generate loss-of-function mutations at targeted sites through error-prone non-homologous end-joining, the addition of a homologous DNA template permits the introduction of defined sequences at desired sites through homology-directed repair (HDR). Cas9-mediated HDR has enabled precise genomic editing in cell lines and organoids, and HDR has also been used in mice to introduce reporter genes, correct disease alleles, and introduce targeted point mutations in several adult tissues^4,12–17^.

By circumventing the need to generate a new genetically engineered mouse line for every oncogenic allele of interest, Cas9-mediated HDR has the potential to provide an enormous decrease in both the time and cost associated with modeling cancers with defined oncogenic driver mutations in vivo. Here, we report a method for efficient de novo cancer modeling in mice using AAV/Cas9-mediated HDR. We demonstrate that this approach enables multiplexed somatic HDR to produce genetically diverse tumors within individual mice. Furthermore, while current autochthonous cancer models rely on semiquantitative techniques to assess tumor burden, we describe an HDR-based DNA barcoding strategy that allows for quantitative tumor analyses using high-throughput sequencing. The integration of AAV/Cas9-mediated HDR with tumor barcoding and deep sequencing represents a novel platform to rapidly and quantitatively interrogate the function of multiple precise mutations simultaneously in mouse models of diverse human cancer types.

## Results

### AAV/Cas9-mediated HDR enables alteration of endogenous *Kras*

**Fig. 1:**
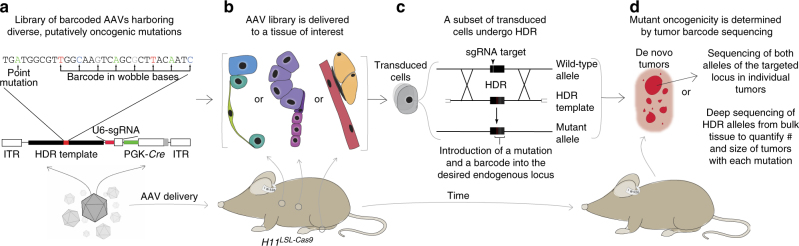
A cancer modeling platform that integrates AAV/Cas9-mediated somatic HDR with tumor barcoding and sequencing to enable the rapid introduction and functional investigation of putative oncogenic point mutations in vivo. **a**–**d** Schematic of the pipeline to quantitatively measure the in vivo oncogenicity of a panel of defined point mutations. **a** A library of AAV vectors is generated such that each AAV contains: (1) a template for homology-directed repair (HDR) containing a putatively oncogenic point mutation and a random DNA barcode encoded in the adjacent wobble bases; (2) an sgRNA targeting the desired endogenous locus to enhance HDR; and (3) *Cre*-recombinase to activate a conditional *Cas9* allele (*H11* ^*LSL-Cas9*^) and other Cre-dependent alleles in genetically engineered mice. **b** The AAV library is delivered to a tissue of interest. **c** Following transduction, a subset of cells undergo AAV/Cas9-mediated HDR in which the locus of interest is cleaved by Cas9 at the sgRNA target site and repaired using the AAV HDR template. This results in the introduction of the desired point mutation and a unique DNA barcode at the targeted locus. **d** Somatic cells engineered with a point mutation may develop into de novo tumors if the introduced mutation is sufficient to initiate tumorigenesis and drive tumor growth. Two independent approaches can be used to analyze tumors: (1) the targeted region in individual tumors can be sequenced to characterize both alleles of the targeted gene, or (2) next-generation sequencing of the targeted region can be used to determine the number, size, and genotype of each tumor directly from bulk tissue in a quantitative and multiplexed manner

**Fig. 2:**
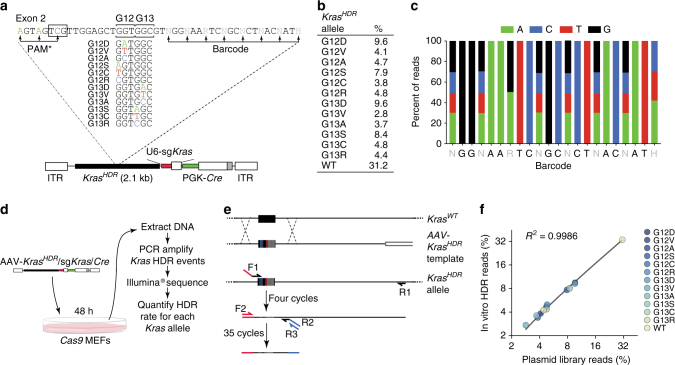
Design and validation of an AAV targeting vector library to introduce all *Kras* codon 12 and 13 single-nucleotide non-synonymous point mutations into somatic mouse cells in a multiplexed manner. **a** AAV vector pool for Cas9-mediated HDR into the endogenous *Kras* locus (AAV-*Kras* ^*HDR*^/sg*Kras*/*Cre*). Each vector contained an HDR template with 1 of 12 non-synonymous *Kras* mutations at codons 12 and 13 (or wild-type *Kras*), silent mutations within the PAM (boxed sequence) and sgRNA homology region (PAM*), and a random 8-nucleotide barcode within the wobble positions of the adjacent codons for stable DNA barcoding of individual tumors. **b** Representation of each *Kras* ^*HDR*^ allele in the AAV-*Kras* ^*HDR*^/sg*Kras*/*Cre* plasmid library. **c** Diversity of the barcode region in the AAV-*Kras* ^*HDR*^/sg*Kras*/*Cre* plasmid library. **d** Schematic of the experiment to test for HDR bias. A Cas9-expressing cell line was transduced with AAV-*Kras* ^*HDR*^/sg*Kras*/*Cre* and *Kras* ^*HDR*^ alleles were sequenced to quantify HDR events. **e** Schematic of the PCR strategy to specifically amplify *Kras* ^*HDR*^ alleles introduced into the genome via HDR. Forward primer 1 (F1) binds to the sequence containing the 3 PAM* mutations, while reverse primer 1 (R1) binds the endogenous *Kras* locus, outside the sequence present in the homology arm of the *Kras* ^*HDR*^ template. F2 binds to the Illumina adaptor added by F1, R2 binds to a region near exon 2, and R3 binds to the Illumina adapter added in the same reaction by R2. **f** Frequency of HDR events for each *Kras* ^*HDR*^ allele plotted against the initial frequency of each *Kras* mutant allele in the AAV-*Kras* ^*HDR*^/sg*Kras*/*Cre* plasmid library. High correlation between the initial plasmid library and the representation of mutant *Kras* alleles following HDR suggests little to no HDR bias

To investigate the oncogenic function of diverse point mutations in vivo in a quantitative and systematic manner, we developed a platform for somatic AAV/Cas9-mediated HDR that incorporates DNA barcoding and high-throughput sequencing in autochthonous mouse models of several cancer types (Fig. 1). We designed, generated, and validated a library of AAV vectors to introduce all possible *Kras* codon 12 and 13 single-nucleotide non-synonymous point mutations into somatic mouse cells in a multiplexed manner (Fig. 2a–c and Supplementary Fig. 1). Each AAV contained a ~2 kb *Kras* HDR template, a single-guide RNA (sgRNA) targeting the second exon of *Kras* to enhance somatic HDR, and *Cre*-recombinase (AAV-*Kras* ^*HDR*^/sg*Kras*/*Cre*; Fig. 2a and Supplementary Fig. 1a)^18^.

The *Kras* ^*HDR*^ template contained the genomic sequence flanking the second exon of *Kras* and either wild-type (WT) *Kras* exon 2 or exon 2 with one of 12 single-nucleotide non-synonymous mutations in codon 12 or 13 (Fig. 2a). Each *Kras* ^*HDR*^ template also contained silent mutations within the sg*Kras* target sequence and associated protospacer adjacent motif (PAM*) to prevent Cas9/sg*Kras*-mediated cleavage of *Kras* ^*HDR*^ alleles (Fig. 2a and Supplementary Fig. 1b, d). To enable the parallel quantification of individual tumors by high-throughput sequencing of the *Kras* ^*HDR*^ allele in DNA from bulk tissue, we diversified the *Kras* ^*HDR*^ templates with a random barcode engineered into eight wobble positions of the codons downstream of codons 12 and 13 (Fig. 2a and Supplementary Fig. 1b, c).

The AAV vectors also encoded *Cre*-recombinase, which enabled tumor initiation in mice containing a Cre-regulated *Cas9* allele (*H11* ^*LSL-Cas9*^)^19^ (Fig. 2a). Cre-recombinase also led to labeling of transduced cells via a fluorescent Cre-reporter allele (*Rosa26* ^*LSL-Tomato*^)^20^ and enabled deletion of the tumor suppressor genes *p53* or *Lkb1* using floxed alleles^21,22^. We packaged the AAV-*Kras* ^*HDR*^/sg*Kras*/*Cre* library using an AAV8 capsid that enables high titer production, efficient transduction of mouse lung epithelial cells in vivo (Supplementary Fig. 2), and transduction of a wide range of adult mouse tissues^23^.

We initially transduced Cas9-expressing cells in culture with AAV-*Kras* ^*HDR*^/sg*Kras*/*Cre* to determine whether AAV/Cas9-mediated HDR would be an unbiased method to engineer point mutations into the endogenous *Kras* locus (Fig. 2d). We developed a PCR strategy to specifically amplify *Kras* ^*HDR*^ alleles, thus avoiding amplification of the endogenous *Kras* locus or any residual episomal AAV-*Kras* ^*HDR*^/sg*Kras*/*Cre* (Fig. 2e). High-throughput sequencing of the amplified *Kras* ^*HDR*^ regions from transduced cells confirmed the generation of all point mutant *Kras* alleles. The representation of *Kras* ^*HDR*^ alleles in these cells was highly correlated with their representation in the plasmid library, suggesting that HDR using our AAV vector was not discernably biased by any single-nucleotide *Kras* codon 12 or 13 point mutation in the *Kras* ^*HDR*^ template (Fig. 2f). Therefore, any differential expansion of tumors harboring specific *Kras* mutant alleles in vivo could be attributed to differences in the oncogenicity of Kras variants rather than HDR bias.

### Somatic HDR initiates mutant Kras-driven lung tumors in vivo

**Fig. 3:**
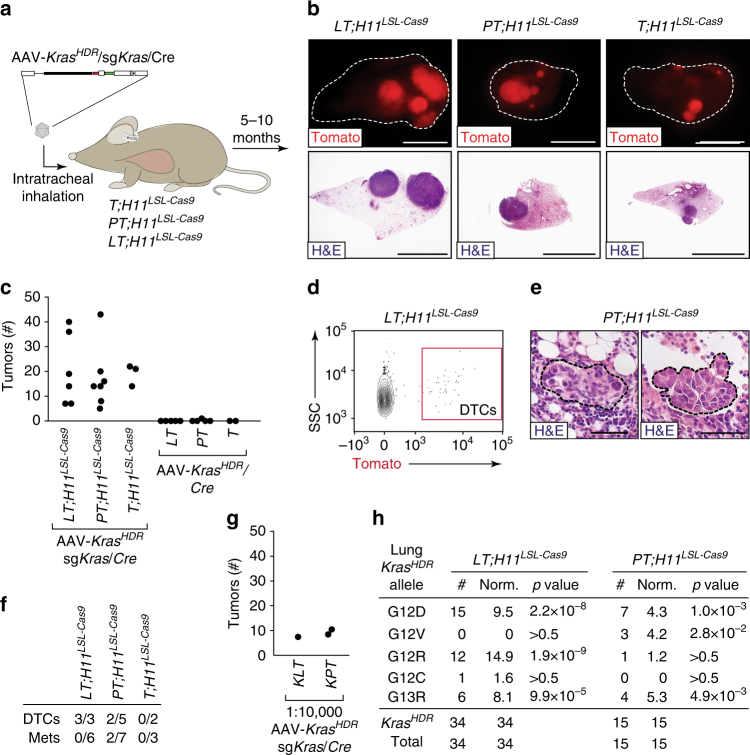
AAV/Cas9-mediated somatic HDR initiates oncogenic Kras-driven lung tumors. **a** Schematic of the experiment to introduce point mutations and a DNA barcode into the endogenous *Kras* locus of lung epithelial cells in *Rosa26* ^*LSL-Tomato*^ *;H11* ^*LSL-Cas9*^ (*T;H11* ^*LSL-Cas9*^), *p53* ^*flox/flox*^ *;T;H11* ^*LSL-Cas9*^ (*PT;H11* ^*LSL-Cas9*^), and *Lkb1* ^*flox/flox*^ *;T;H11* ^*LSL-Cas9*^ (*LT;H11* ^*LSL-Cas9*^) mice by intratracheal administration of AAV-*Kras* ^*HDR*^/sg*Kras*/*Cre* (8.4 × 10^10^ vector genomes per mouse). **b** Representative fluorescence and histology images of Tomato^positive^ lung tumors in *LT;H11* ^*LSL-Cas9*^, *PT;H11* ^*LSL-Cas9*^, and *T;H11* ^*LSL-Cas9*^ mice transduced with AAV-*Kras* ^*HDR*^/sg*Kras*/*Cre*. Scale bars = 5 mm. **c** Quantification of lung tumors in the indicated genotypes of mice transduced with the indicated AAV vectors (with and without sg*Kras*). Each dot represents one mouse. **d** Representative FACS plot showing Tomato^positive^ DTCs in the pleural cavity of an *LT;H11* ^*LSL-Cas9*^ mouse with AAV-*Kras* ^*HDR*^/sg*Kras*/*Cre*-initiated lung tumors. Plot shows forward scatter/side scatter (SSC)-gated viable cancer cells (DAPI/CD45/CD31/F4-80/Ter119^negative^). **e** Histology of lymphatic metastases from AAV-*Kras* ^*HDR*^/sg*Kras*/*Cre*-initiated lung tumors in *PT;H11* ^*LSL-Cas9*^ mice. Scale bar = 50 µm. **f** Number of AAV-*Kras* ^*HDR*^/sg*Kras*/*Cre*-transduced mice of each genotype that had disseminated tumors cells in their pleural cavity (DTCs > 10) or metastases out of the total number of mice analyzed. **g** *Kras* ^*LSL-G12D*/+^ *;LT* (*KLT*) and *Kras* ^*LSL-G12D*/+^ *;PT* (*KPT*) mice transduced with a 1:10,000 dilution of AAV-*Kras* ^*HDR*^/sg*Kras*/*Cre* developed approximately half as many tumors as the *PT;H11* ^*LSL-Cas9*^ and *LT;H11* ^*LSL-Cas9*^ mice transduced with undiluted virus. If all *Kras* ^*HDR*^ alleles in the AAV-*Kras* ^*HDR*^/sg*Kras*/*Cre* library are oncogenic, this suggests that AAV/Cas9-mediated HDR occurs in ~0.02% of transduced cells. Alternatively, if only 20% of the mutant alleles in the AAV-*Kras* ^*HDR*^/sg*Kras*/*Cre* library are oncogenic, this suggests that HDR occurs in ~0.1% of transduced cells. **h** Diverse HDR-generated oncogenic *Kras* alleles in individual lung tumors dissected from *LT;H11* ^*LSL-Cas9*^ and *PT;H11* ^*LSL-Cas9*^ mice transduced with AAV-*Kras* ^*HDR*^/sg*Kras*/*Cre*. Number of tumors with each allele is indicated. Alleles that were not identified in any lung tumors are not shown. For the *Kras* ^*HDR*^ alleles identified, Bonferroni corrected *p* values for likelihood of enrichment relative to WT are shown (Fisher’s exact test generalized for structural zeros; see Methods section)

To determine whether HDR in somatic cells could initiate tumors, and to investigate whether *Kras* variants differ in their ability to drive tumorigenesis, we delivered the AAV-*Kras* ^*HDR*^/sg*Kras*/*Cre* library intratracheally to the lungs of mice with the *H11* ^*LSL-Cas9*^ allele (Fig. 3a and Supplementary Fig. 3a). Three different genotypes of mice were transduced to provide additional insight into whether concurrent inactivation of tumor suppressor genes modulates *Kras* variant oncogenicity: (1) *Rosa26* ^*LSL-Tomato*^;*H11* ^*LSL-Cas9*^ (*T*;*H11* ^*LSL-Cas9*^) mice, (2) *p53* ^*flox*/*flox*^;*T*;*H11* ^*LSL-Cas9*^ (*PT*;*H11* ^*LSL-Cas9*^) mice in which tumors would lack p53, and (3) *Lkb1* ^*flox*/*flox*^;*T*;*H11* ^*LSL-Cas9*^ (*LT*;*H11* ^*LSL-Cas9*^) mice in which tumors would lack Lkb1 (Fig. 3a and Supplementary Fig. 3a).

*LT*;*H11* ^*LSL-Cas9*^ mice were the first to show signs of tumor development including tachypnea and weight loss between 5 and 7 months after AAV administration. This is consistent with the rapid growth of lung tumors initiated in mice with a Cre-regulated *Kras* ^*G12D*^ allele and floxed *Lkb1* alleles^24–26^. *LT*;*H11* ^*LSL-Cas9*^ mice had many large primary lung tumors, resulting in high tumor burden (Fig. 3b, c and Supplementary Fig. 3b–d). Histological analysis confirmed the presence of large adenomas and adenocarcinomas in these mice (Fig. 3b and Supplementary Fig. 3b). *PT*;*H11* ^*LSL-Cas9*^ mice also developed numerous large primary lung tumors 6–8 months after viral transduction. Compared to the *LT*;*H11* ^*LSL-Cas9*^ mice, tumors initiated in *PT*;*H11* ^*LSL-Cas9*^ mice had more pronounced nuclear atypia, a characteristic feature of p53 deficiency^27^. Finally, *T*;*H11* ^*LSL-Cas9*^ mice developed smaller, less histologically advanced lesions, even at later time points (>10 months after tumor initiation; Fig. 3b, c and Supplementary Fig. 3b–d). Mice transduced with a 10-fold lower titer of AAV-*Kras* ^*HDR*^/sg*Kras*/*Cre* developed proportionally fewer tumors (Supplementary Fig. 3e). Importantly, delivery of an analogous AAV vector without sg*Kras* (AAV-*Kras* ^*HDR*^/*Cre*) to *T*, *PT*, and *LT* mice did not lead to efficient tumor initiation (Fig. 3c and Supplementary Fig. 4). This indicates that neither *p53* nor *Lkb1* deficiency, combined with high-level AAV vector transduction, is sufficient to drive lung tumorigenesis. Several *LT*;*H11* ^*LSL-Cas9*^ and *PT*;*H11* ^*LSL-Cas9*^ mice with AAV-*Kras* ^*HDR*^/sg*Kras*/*Cre*-initiated tumors developed invasive primary lung tumors, had disseminated tumor cells (DTCs) in their pleural cavities, and had lymph node metastases (Fig. 3d–f). Thus, AAV-*Kras* ^*HDR*^/sg*Kras*/*Cre*-induced tumors can progress into malignant and metastatic lung cancer.

To approximate the efficiency of somatic HDR in lung epithelial cells in vivo, we compared the number of lung tumors initiated by AAV-*Kras* ^*HDR*^/sg*Kras*/*Cre* in mice containing the *H11* ^*LSL-Cas9*^allele (in which Kras would be activated by HDR) to the number of lung tumors initiated in *Kras* ^*LSL-G12D*^ mice (in which Kras would be activated by Cre; Fig. 3g). We transduced *Kras* ^*LSL-G12D*/+^ *;PT* and *Kras* ^*LSL-G12D*/+^ *;LT* mice with a 1:10,000 dilution of AAV-*Kras* ^*HDR*^/sg*Kras*/*Cre* (Fig. 3g). These mice developed approximately half as many tumors as mice in which oncogenic *Kras* alleles were generated by AAV/Cas9-mediated somatic HDR after transduction of lung epithelial cells with undiluted AAV-*Kras* ^*HDR*^/sg*Kras*/*Cre* (Fig. 3g). This result is consistent with an HDR frequency between 0.02 and 0.1% (Fig. 3g). Importantly, this rate of HDR combined with the high-transduction efficiency of AAV enables the parallel initiation of numerous lung tumors within individual mice (Fig. 3c).

To determine whether tumors initiated using AAV-*Kras* ^*HDR*^/sg*Kras*/*Cre* harbored mutant *Kras* ^*HDR*^ alleles, we analyzed the *Kras* locus in cancer cells from large, individual lung tumors from *LT*;*H11* ^*LSL-Cas9*^ and *PT*;*H11* ^*LSL-Cas9*^ mice (Fig. 3c). PCR amplification using primers specific to the *Kras* ^*HDR*^ allele confirmed the presence of an oncogenic *Kras* allele with a unique barcode in each tumor (Figs. 2e, 3h). Interestingly, only five *Kras* variants were identified in ~50 large lung tumors, consistent with differential selection of *Kras* variants in lung tumorigenesis (Fig. 3h).

By analyzing individual tumors, we were able to carefully assess the *Kras* ^*HDR*^ allele as well as the second *Kras* allele in each tumor (Supplementary Fig. 5). Approximately half of the oncogenic *Kras* ^*HDR*^ alleles resulted from perfect HDR events in which a *Kras* point mutation and a unique barcode were seamlessly recombined into the endogenous *Kras* locus. The remaining *Kras* ^*HDR*^ alleles were seamless from the 5′ end through mutant exon 2, but contained small duplications, insertions, or deletions in intron 2 (Supplementary Fig. 5). Importantly, none of these alterations would be expected to disrupt splicing from mutant exon 2 into exon 3. Additionally, almost all tumors harbored Cas9-induced indels in the second *Kras* allele, which is consistent with the loss of the wild-type *KRAS* allele observed in some oncogenic KRAS-driven human tumors (Supplementary Fig. 6)^28,29^. While previous studies have documented enhanced Kras^G12D^- and Kras^Q61L^-driven lung tumor growth following inactivation of the wild-type *Kras* allele in mice^30,31^, our results suggest that wild-type Kras may suppress the growth of lung tumors driven by many different oncogenic Kras variants.

### Somatic HDR can initiate pancreatic cancer

In addition to driving human lung cancer, oncogenic *KRAS* mutations are nearly ubiquitous in human pancreatic ductal adenocarcinoma (PDAC)^32^. Furthermore, genomic and clinical data from PDAC patients indicate that KRAS variants differentially impact downstream signaling as well as patient prognoses^32,33^. Expression of *Kras* ^*G12D*^ or *Kras* ^*G12V*^ in pancreatic cells of genetically engineered mice drives widespread formation of pancreatic intra-epithelial neoplasias (PanINs), but rarely leads to PDAC within 1 year^32,34,35^. However, *Kras* ^*G12D*^ or *Kras* ^*G12V*^ expression in mouse pancreatic cells combined with *p53* mutation—a frequent event in human PDAC—reliably leads to the development of PDAC^35^.

**Fig. 4:**
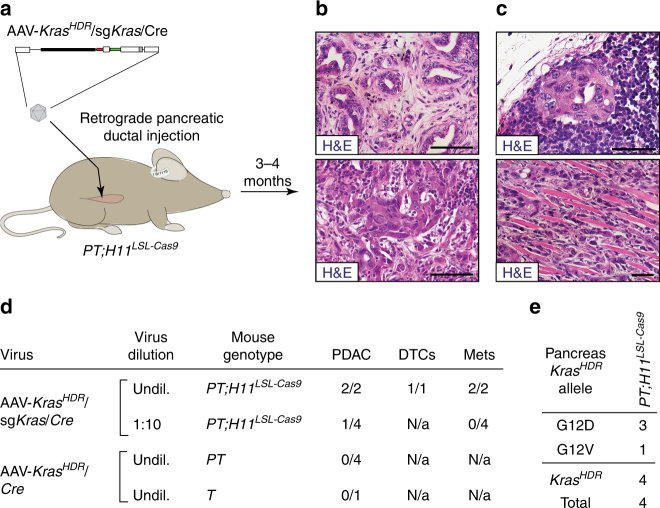
Introduction of mutant *Kras* variants into pancreatic cells using AAV/Cas9-mediated HDR drives the formation of metastatic PDAC. **a** Schematic of retrograde pancreatic ductal injection of AAV-*Kras* ^*HDR*^/sg*Kras*/*Cre* (∼1.7 × 10^11^ vector genomes per mouse) into *PT;H11* ^*LSL-Cas9*^ mice to induce pancreatic cancer. **b** Histology of pancreatic tumors initiated by retrograde pancreatic ductal injection of AAV-*Kras* ^*HDR*^/sg*Kras*/*Cre* into *PT;H11* ^*LSL-Cas9*^ mice. Scale bars = 75 µm. **c** Histology of metastases in the lymph node (upper panel) and diaphragm (lower panel) in *PT;H11* ^*LSL-Cas9*^ mice with PDAC. Scale bars = 50 µm. **d** Incidence of PDAC, DTCs in the peritoneal cavity, and metastases in the indicated genotypes of mice (shown as the number of mice with cancer, DTCs, or metastases out of the total number of mice analyzed), 3–13 months after transduction with either AAV-*Kras* ^*HDR*^/sg*Kras*/*Cre* or AAV-*Kras* ^*HDR*^/*Cre* (∼2.9 × 10^11^ vector genomes per mouse). AAV-*Kras* ^*HDR*^/sg*Kras*/*Cre* was administered at the stock concentration (“undil.”) or at a 1:10 dilution (“1:10”). **e** HDR-generated oncogenic *Kras* alleles in individually dissected pancreatic tumor masses. Number of tumors with each allele is indicated. Alleles that were not identified in any pancreatic tumor masses are not shown

Thus, to determine whether AAV/Cas9-mediated somatic HDR could also induce cancer-initiating oncogenic point mutations in pancreatic epithelial cells, we transduced *PT*;*H11* ^*LSL-Cas9*^ mice with AAV-*Kras* ^*HDR*^/sg*Kras*/*Cre* by retrograde pancreatic ductal injection (Fig. 4a and Supplementary Fig. 7a)^19^. These mice developed PanINs as well as PDAC (Fig. 4b and Supplementary Fig. 7b, c). Several mice also developed metastatic PDAC, consistent with the aggressive nature of the human disease (Fig. 4c, d and Supplementary Fig. 7d, e). Sequencing of *Kras* ^*HDR*^ alleles from several individual pancreatic tumor masses uncovered oncogenic *Kras* ^*G12D*^ and *Kras* ^*G12V*^ alleles with unique barcodes (Fig. 4e). Consistent with the requirement for oncogenic Kras to initiate PDAC, transduction of pancreatic cells in *PT* mice by retrograde pancreatic ductal injection of our negative control AAV-*Kras* ^*HDR*^/*Cre* vector did not induce any pancreatic tumors (Fig. 4d).

### HDR in somatic skeletal muscle cells induces sarcoma

The history of the RAS oncogenes began with their identification as the transforming factors in retroviruses capable of inducing rat sarcoma (hence, RAS)^36^. Human sarcomas are a highly heterogeneous cancer type comprised of over 50 histological subtypes that arise from many tissue and cell types^37^. Soft tissue sarcomas harbor diverse genomic alterations, including recurrent mutations in the tumor suppressor *TP53* as well as the RAS pathway components *NF1*, *PTEN*, and *PIK3CA* ^38,39^. Although *KRAS* is infrequently mutated in sarcomas, *KRAS* mutations occur in undifferentiated pleomorphic sarcomas as well as in rhabdomyosarcomas, which account for nearly half of pediatric soft tissue sarcomas^37,40–42^. Importantly, sarcomas have been induced in genetically engineered mouse models through the expression of *Kras* ^*G12D*^ and inactivation of *p53* ^37,38,43,44^. However, generating mouse models of soft tissue sarcoma remains a challenge given the genetic diversity of the disease, and methods to rapidly model oncogene mutations in sarcomagenesis have not been described^37,44^.

**Fig. 5:**
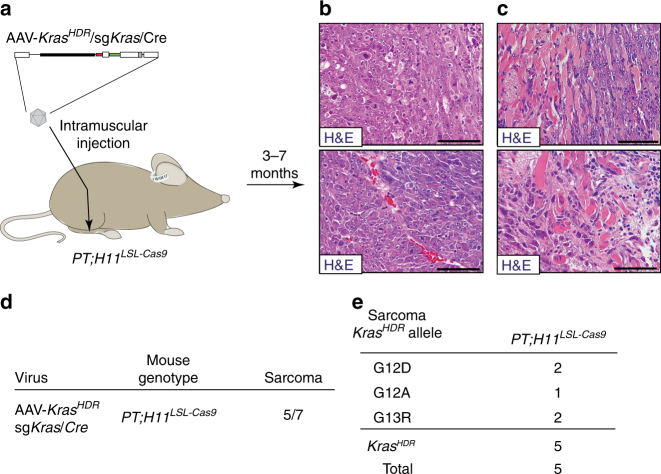
Introduction of mutant *Kras* variants into muscle cells using AAV/Cas9-mediated HDR induces invasive sarcoma. **a** Schematic of intramuscular administration of AAV-*Kras* ^*HDR*^/sg*Kras*/*Cre* (1.6 × 10^11^ vector genomes per mouse) into the gastrocnemii of *PT;H11* ^*LSL-Cas9*^ mice to induce sarcomas. **b**, **c** Histology of stereotypical sarcoma (**b**) and invasive sarcoma (**c**) initiated by intramuscular administration of AAV-*Kras* ^*HDR*^/sg*Kras*/*Cre* into the gastrocnemii of *PT;H11* ^*LSL-Cas9*^ mice. Scale bars = 75 µm. **d** Sarcoma incidence in *PT;H11* ^*LSL-Cas9*^ mice 3–7 months after intramuscular administration of AAV-*Kras* ^*HDR*^/sg*Kras*/*Cre*. Incidence represents the number of mice that developed sarcomas out of the total number of mice injected. **e** HDR-generated oncogenic *Kras* alleles in sarcomas. Number of tumors with each allele is indicated. Alleles not identified in any sarcomas are not shown

To determine whether AAV/Cas9-mediated somatic HDR could be used to introduce point mutations into *Kras* and drive sarcoma formation, we performed intramuscular injections of AAV-*Kras* ^*HDR*^/sg*Kras*/*Cre* into the gastrocnemii of *PT*;*H11* ^*LSL-Cas9*^ mice (Fig. 5a and Supplementary Fig. 8a). These mice developed rapidly growing and invasive skeletal muscle sarcomas that harbored uniquely barcoded oncogenic *Kras* ^*G12D*^, *Kras* ^*G12A*^, or *Kras* ^*G13R*^ alleles (Fig. 5b–e and Supplementary Fig. 8b–d). The successful application of this platform for modeling tumorigenesis in diverse tissues highlights its broad applicability for multiplexed functional analyses of oncogenic driver mutations in a wide range of cancer types.

### Tumor barcode sequencing reveals Kras variant oncogenicity

**Fig. 6:**
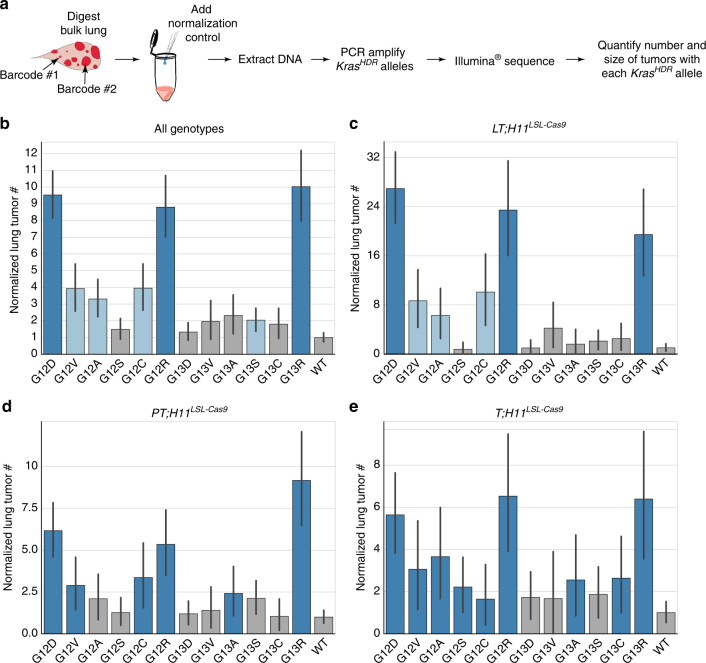
Multiplexed, quantitative analysis of *Kras* mutant oncogenicity using AAV/Cas9-mediated somatic HDR and high-throughput sequencing of barcoded lung tumors. **a** Pipeline to quantitatively determine the number, size, and genotype of individual tumors directly from bulk lung samples by high-throughput sequencing of tumor barcodes. **b**–**e** Number of lung tumors harboring each mutant *Kras* allele normalized to its initial representation (mutant representation in the AAV plasmid library divided by WT representation in the AAV plasmid library) and relative to WT (mutant tumor # divided by WT tumor #). Variants present in significantly more tumors than WT (two-sided Fisher’s exact test; *p* < 0.05) are colored blue; darker blue indicates no significant difference from G12D (*p* > 0.05), lighter blue indicates significantly less tumors with the indicated variant than G12D (*p* < 0.05). Error bars are bootstrapped 95% confidence intervals. Bar plots were generated from pooled data from all mouse genotypes (*N* = 15) (**b**), or individually from *LT;H11* ^*LSL-Cas9*^ (*N* = 6) (**c**), *PT;H11* ^*LSL-Cas9*^ (*N* = 6) (**d**), or *T;H11* ^*LSL-Cas9*^ (*N* = 3) (**e**) mice. Note that different *y* axis scales are used in each plot

Whereas conventional methods to assess the effects of different genomic alterations in autochthonous cancer models largely rely on manual quantification of tumor number and size, we recently established a simple yet high-throughput approach to determine the genotype and number of neoplastic cells within individual tumors directly from bulk tissue by tumor barcoding and sequencing (Tuba-seq)^26^. Here, we extended the Tuba-seq platform to enable multiplexed and quantitative measurements of HDR-initiated tumors in vivo by engineering a random barcode into the wobble bases adjacent to an activating point mutation of interest. Since neoplastic cells within AAV-*Kras* ^*HDR*^/sg*Kras*/*Cre*-initiated tumors each contain a copy of the same unique DNA barcode, the relative number of neoplastic cells within each tumor can be determined by deep sequencing of the barcode region (Figs. 1d, 6a). Furthermore, by adding a normalization control consisting of a known number of cells with a known barcode to each bulk tissue sample prior to sequencing, the absolute number of neoplastic cells in each tumor can be estimated from relative sequencing read counts^26^. To determine the *Kras* genotype and number of neoplastic cells in each tumor in *T*;*H11* ^*LSL-Cas9*^, *PT*;*H11* ^*LSL-Cas9*^, and *LT;H11* ^*LSL-Cas9*^ mice, we added a normalization control consisting of DNA from 5 × 10^5^ cells with a known barcode to each lung sample (Fig. 6a and Supplementary Fig. 9). We then extracted DNA from each sample, PCR-amplified the *Kras* ^*HDR*^ alleles, and deep-sequenced the variant-barcode region (Fig. 6a and Supplementary Fig. 9).

Following high-throughput sequencing, we corrected for recurrent sequencing errors and the possibility of individual tumors having identical barcodes (see Methods section). We then estimated the absolute number of neoplastic cells in each tumor by normalizing tumor barcode-sequencing read counts to the number of reads from the normalization control DNA. This analysis pipeline was exceptionally reproducible with a high degree of concordance in tumor sizes across technical replicates (Supplementary Fig. 10).

High-throughput sequencing of the *Kras* ^*HDR*^ variant-barcode region uncovered many AAV-*Kras* ^*HDR*^/sg*Kras*/*Cre*-induced lung tumors in *T*;*H11* ^*LSL-Cas9*^, *PT*;*H11* ^*LSL-Cas9*^, and *LT;H11* ^*LSL-Cas9*^ mice (Supplementary Fig. 11 and Supplementary Data 1). Normalizing tumor number to the initial representation of each *Kras* ^*HDR*^ allele in the AAV-*Kras* ^*HDR*^/sg*Kras*/*Cre* vector library allowed us to directly compare the in vivo oncogenicity of each Kras variant (Fig. 6b–e, Supplementary Fig. 11d, e, and Supplementary Data 2). Across >500 tumors, *Kras* ^*G12D*^ was the most common variant, consistent with *KRAS* ^*G12D*^ being the most frequent *KRAS* mutation in human lung adenocarcinoma in never-smokers^45^. *Kras* ^*G12A*^, *Kras* ^*G12C*^, and *Kras* ^*G12V*^—the next most frequent *KRAS* variants in lung adenocarcinoma in never-smokers after *KRAS* ^*G12D*^—were identified as moderate drivers of lung tumorigenesis, but were present in fewer tumors than *Kras* ^*G12D*^ (Fig. 6b–e and Supplementary Fig. 11a–e). Interestingly, *Kras* ^*G12R*^ and *Kras* ^*G13R*^ were also identified as potent oncogenic variants, despite being infrequently mutated in human lung cancer (Fig. 6b–e and Supplementary Fig. 11a–e).

*Kras* expression can be influenced by overall changes in codon usage^46,47^. As our barcoding approach changed up to eight codons, we investigated whether alterations in *Kras* codon usage influenced tumor size. By calculating the relative change in codon usage introduced by each unique barcode, we found that the degree to which overall codon usage increased or decreased did not correlate with tumor size (Supplementary Fig. 12)^48^. There was also no bias toward increased or decreased codon usage in the tumors identified (Supplementary Fig. 12).

### Kras oncogenicity is robust to tumor suppressor inactivation

We initiated tumors in *LT;H11* ^*LSL-Cas9*^, *PT*;*H11* ^*LSL-Cas9*^, and *T*;*H11* ^*LSL-Cas9*^ mice to directly assess whether concurrent tumor suppressor alterations modulate the ability of different Kras variants to initiate and drive tumor growth. In general, Lkb1-deficient tumors from *LT;H11* ^*LSL-Cas9*^ mice grew much more rapidly than tumors in *PT*;*H11* ^*LSL-Cas9*^ and *T*;*H11* ^*LSL-Cas9*^ mice (Supplementary Fig. 3a–d). Surprisingly, no statistically significant pairwise deviations in tumor number were observed for any *Kras* variant across tumors with different tumor suppressor genotypes. However, by considering the aggregate deviations in *Kras* variants across genotypes, we observed subtle differences in the spectrum of *Kras* variants in Lkb1-deficient tumors compared to p53-deficient or otherwise wild-type tumors (Supplementary Fig. 11f). These data are consistent with a model in which the strength of signaling induced by these oncogenic Kras variants in vivo is insufficient to engage the p53 pathway; thus, while p53 functions to constrain tumor progression, it does not appear to limit the initial expansion of tumors with different *Kras* genotypes^49^. Additionally, while Lkb1 deficiency increased tumor growth, in general, Lkb1 deficiency did not appear to preferentially synergize with any specific oncogenic Kras variants (Fig. 6c–e and Supplementary Figs. 3d and 11a–e).

### Tuba-seq enables mapping of pancreatic tumors and metastases

**Fig. 7:**
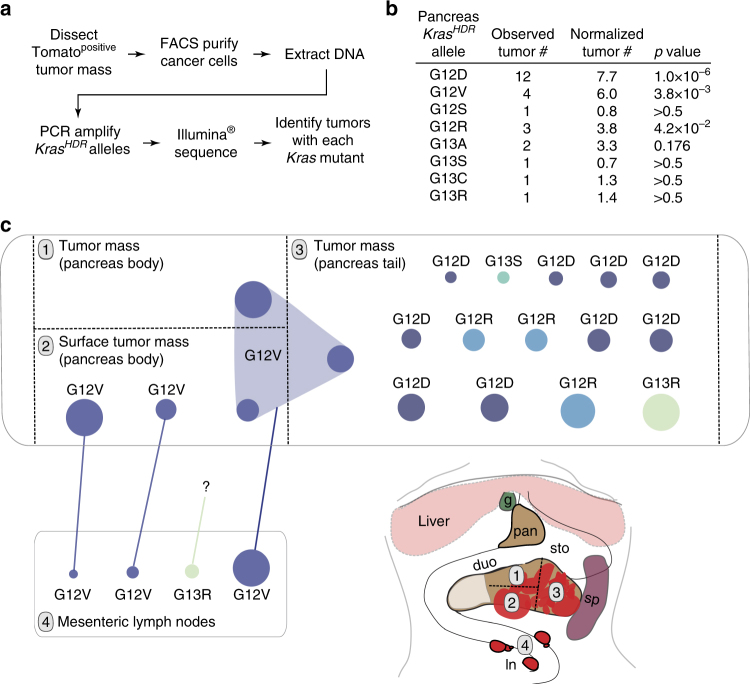
High-throughput sequencing of pancreatic tumor masses enables spatial mapping of tumor clones and phylogenetic tracking of metastases. **a** Analysis pipeline to identify *Kras* ^*HDR*^ alleles in AAV-*Kras* ^*HDR*^/sg*Kras*/*Cre*-initiated tumor masses within the pancreata of *PT;H11* ^*LSL-Cas9*^ mice. **b** Diverse HDR-generated *Kras* alleles identified by tumor barcode sequencing of pancreatic tumor masses from three *PT;H11* ^*LSL-Cas9*^ mice. Numbers of uniquely barcoded primary tumors with each allele (including those identified by individual tumor analyses, as shown in Fig. 4e) are indicated. Alleles not identified in any pancreas tumor masses are not shown. For the *Kras* ^*HDR*^ alleles identified, Bonferroni-corrected *p* values for likelihood of enrichment relative to WT are shown (Fisher’s exact test generalized for structural zeros; see Methods section) (*p* values <0.05 are bold). **c** Multi-region sequencing of a large pancreatic tumor mass in a *PT;H11* ^*LSL-Cas9*^ mouse transduced with AAV-*Kras* ^*HDR*^/sg*Kras*/*Cre* revealed a diverse spectrum of mutant *Kras* alleles and uncovered relationships between primary tumors and their metastatic descendants. Each dot represents a tumor with the indicated *Kras* variant and a unique barcode within each sample (labeled 1–4). Dots connected across different primary tumor samples (labeled 1–3) shared the same *Kras* variant-barcode pair, and are thus presumably regions of the same primary tumor that were present in multiple samples. A colored line links primary tumors and lymph node metastases harboring the same *Kras* variant-barcode pair, indicating a clonal relationship. The size of each dot is scaled according to the size of the tumor that it represents (diameter of the dot = relative size^1/2^). Since the size of pancreatic tumors was not normalized to a control, tumor sizes can only be compared to other tumors within the same sample. Thus, the largest tumors within each sample have been scaled to the same standard size. g gallbladder, sto stomach, duo duodenum, pan pancreas, sp spleen, ln mesenteric lymph nodes

Since our tumor barcoding and sequencing platform (Tuba-seq^26^) allowed us to characterize many individual tumors in parallel from bulk lungs, we anticipated that we could also use this approach to overcome the challenge of identifying and analyzing individual pancreatic tumor clones in multifocal tumor masses initiated in autochthonous mouse models^19^. Therefore, we analyzed bulk pancreatic tumor samples from *PT*;*H11* ^*LSL-Cas9*^ mice transduced with AAV-*Kras* ^*HDR*^/sg*Kras*/*Cre* (Fig. 7a and Supplementary Fig. 13a). Barcode sequencing of pancreatic tumor masses uncovered multiple primary tumor clones per mouse, each harboring a *Kras* ^*HDR*^ allele with a unique DNA barcode (Fig. 7b and Supplementary Data 3). Pancreatic tumors demonstrated oncogenic *Kras* allele preferences with *Kras* ^*G12D*^, *Kras* ^*G12V*^, and *Kras* ^*G12R*^ being significantly enriched (Fisher’s exact test; Fig. 7b). Notably, these three *Kras* variants are also the most prevalent oncogenic *KRAS* mutations in human PDAC^50^.

In addition to determining the in vivo oncogenicity of specific *Kras* variants, our barcode-sequencing approach allowed us to identify contiguous tumor clones from multi-region sequencing of PDAC masses (Fig. 7c and Supplementary Fig. 13b). Finally, using barcode sequencing we were able to uncover clonal relationships between primary tumors and their metastatic descendants (Fig. 7c and Supplementary Fig. 13b).

## Discussion

Prior to the introduction of massively parallel sequencing, one of the most profound challenges in the field of cancer biology was the identification of genomic alterations in human cancers^51^. Over the past decade, next-generation sequencing technologies have been leveraged to catalog millions of somatic mutations in human cancer^52^. With this quantum leap in our understanding of the genomic landscape of human tumors, the burden of progress now rests squarely on our ability to uncover the biological function of cancer-associated mutations. The increasing need for faster, more economical, and higher-throughput functional genomics strategies has found a measure of relief with the emergence of CRISPR/Cas9-based genome-editing technologies. The ability to directly modify the genomes of somatic cells in adult mice represents a substantial advance in cancer modeling, enabling the interrogation of gene function in de novo tumors at only a fraction of the time and cost of conventional systems^4,12^.

Methods to deliver Cas9 and a sgRNA to somatic cells, including transfection and viral transduction techniques, facilitate the generation of tumors harboring putative tumor suppressor gene alterations that have yet to be functionally studied in vivo^12^. The delivery of pools of sgRNAs to introduce multiple somatic loss-of-function mutations in parallel represents another critical milestone on the path to more rapid and higher-throughput studies of the functional consequences of mutations in cancer^26,53,54^. Here, we add multiplexed AAV/Cas9-mediated somatic HDR to the cancer modeling toolkit, further expanding the realm of cancer genotypes that can be easily studied in vivo (Fig. 1). We demonstrate that somatic HDR is a quantitative, scalable, and modular approach for systematic studies of the functional consequences of panels of oncogene mutations in parallel. Using this approach, we rapidly modeled tumors of multiple genotypes in several diverse tissues in vivo. Importantly, this method can be readily integrated with conventional genetically engineered mouse models including those with floxed and reporter alleles. Additionally, as genome engineering strategies continue to facilitate higher-content functional screens in autochthonous cancer models, the lack of correspondingly high-throughput methods for tumor analysis has emerged as a fundamental bottleneck in the functional annotation of cancer genomics data. To hurdle this barrier, we integrated a DNA barcoding strategy into our somatic HDR platform to enable the use of next-generation sequencing to determine the number, size, and genotype of tumors directly from bulk tissue in a quantitative and multiplexed manner (Fig. 1).

Using this HDR platform, we investigated the functional consequences of *KRAS* point mutations, which are among the most common driving events in human cancer^55^. Our study addresses the fundamental question of whether tumorigenesis driven by specific *KRAS* mutations results in different pathological outcomes—indeed, we identify dramatic differences in the oncogenicity of Kras variants in vivo. The elucidation of these quantitative differences in oncogenic potential should facilitate the reclassification and therapeutic stratification of KRAS-driven tumors based on the functional consequences of specific KRAS variants, and contribute to the renewed effort to understand somatic cancer mutations at the level of the nucleotide rather than the gene.

Biochemical differences between KRAS variants as well as the frequency with which certain nucleotide substitutions in KRAS are generated likely both contribute to the non-uniform distribution of *KRAS* variants in human tumors. In vitro experiments have uncovered biochemical, structural, and cell context-specific differences between KRAS variants that could serve as biological substrates for tumorigenic selection. Notably, 98% of *KRAS* mutations in human tumors affect active site residues, thus limiting GTPase activity^1,55^. However, despite measurable differences in both the intrinsic and GAP-mediated GTPase activity of KRAS variants, neither biochemical property correlates with in vivo oncogenicity (Supplementary Fig. 14)^2^. Oncogenic proclivity was also not well-predicted by affinity for RAF, although variants with relatively low RAF affinities (Kras^G12D^, Kras^G12V^, and Kras^G12R^) tended to be more oncogenic in our models of lung and pancreatic cancer (Supplementary Fig. 14c, f)^2^. Collectively, these data suggest that the in vivo oncogenicity of KRAS variants may be better described either by an alternate biochemical property, or more likely, through the integration of multiple biochemical inputs.

Though the biochemical mechanisms remain unclear, our data indicate that the primary determinant of mutant *KRAS* representation in human PDAC is likely of a biological rather than etiological origin; we identified Kras^G12D^, Kras^G12V^, and Kras^G12R^ as the most potent drivers of PDAC in vivo, which together account for >90% of all *KRAS* mutations in human PDAC (Fig. 7b, Supplementary Fig. 15, and Supplementary Data 4). This conclusion is supported by autochthonous zebrafish models of Kras-induced pancreatic tumorigenesis, and suggests that the cells of origin of PDAC may not be subjected to the same levels of mutagenic bias that afflict tissues such as the lung whose mutational burden can be more directly linked to specific external factors^55–58^.

The forces driving the spectrum of *KRAS* mutations in human lung cancer appear to be less centralized than in cancers of the pancreas. Differences between the oncogenicity of specific Kras variants in our mouse model compared to their prevalence in human lung cancers indicate that factors other than the intrinsic oncogenicity of KRAS mutants likely influence their representation in the human disease. Although *Kras* ^*G12D*^, *Kras* ^*G12C*^, *Kras* ^*G12V*^, and *Kras* ^*G12A*^ were enriched in lung tumors in mice—as they are in humans—*Kras* ^*G12C*^ was less frequent than expected given the spectrum of *KRAS* mutations in the human disease, while *Kras* ^*G12R*^ and *Kras* ^*G13R*^ were more frequent (Fig. 6b, Supplementary Fig. 15, and Supplementary Datas 2 and 4).

The high prevalence of *KRAS* ^*G12C*^ in human lung cancer is largely driven by the mutational processes that lead to lung cancer initiation. Indeed, numerous studies have identified mutational signatures in the lung cancer genome that represent extant records of the tumor’s etiological origins^56,58,59^. By examining the biases associated with tumor-extrinsic mutational signatures^58^—including the smoking-related signature and the molecular clock-like signature—we estimate that *KRAS* ^*G12C*^ is approximately five times more likely than *KRAS* ^*G12D*^ to occur in the cells of origin of lung adenocarcinomas (Supplementary Fig. 16a and Supplementary Data 5). Furthermore, whereas *KRAS* ^*G12C*^ is the most frequent *KRAS* mutation in lung adenocarcinomas in current/former-smokers, it is approximately three times less prevalent in never-smokers in which *KRAS* ^*G12D*^ is the dominant mutation (Supplementary Fig. 16b and Supplementary Data 6)^45,58,59^. These results underscore the importance of a holistic view of mutational prevalence in human cancer with careful consideration for the mechanisms underlying both the formation and function of somatic cancer mutations.

Neither mutational biases nor biochemical properties appear to fully explain why Kras^G12R^ and Kras^G13R^ were identified as potent drivers of lung tumorigenesis in mice but are rare in human lung adenocarcinomas (Fig. 6b, Supplementary Fig. 15, and Supplementary Datas 2 and 4). Existing structural, biochemical, in vitro, and in vivo data might compel one to believe that these two variants are indeed oncogenic. Arginine substitutions at KRAS residue 12 or 13 prevent formation of transition state complexes with GAPs—an interaction that stimulates the minimal intrinsic GTPase activity of wild-type KRAS by approximately five orders of magnitude^1,60^. Kras^G12R^ has a lower GAP-mediated GTPase activity than either Kras^G12C^ or Kras^G12D^ ^1^. *KRAS* ^*G12R*^ expression also leads to cellular transformation in culture, and drives de novo tumor formation in the lung and pancreas in various autochthonous systems (Figs. 6b, 7b)^57,61^. Human cancer genomics data also provides evidence of the oncogenicity of G12R and G13R substitutions in RAS proteins: *KRAS* ^*G12R*^ accounts for >10% of KRAS-driven PDACs (Supplementary Fig. 15c and Supplementary Data 4), while *HRAS* ^*G13R*^ is the fourth most frequent *HRAS* mutation in human cancers and *NRAS* ^*G13R*^ is common in colorectal cancers^1,8,62^. Taken together, these data warrant a shift in perspective from the conundrum of why these variants are so oncogenic in our model to the question of why they are not in people.

The answer may lie with one, or perhaps many, of the myriad variables that define the molecular, cellular, and environmental context within which a tumor forms. A simple explanation could be species-specific effects. Although mouse and human *KRAS* orthologs share 97% (183/188) amino acid identity, the amino acid mismatches are concentrated in the C-terminal hypervariable region (HVR) of KRAS (Supplementary Fig. 17). The RAS HVR regulates the subcellular trafficking and localization, post-translational modification, and effector binding of RAS proteins^1,63^. Additionally, species-specific regulatory mechanisms could differentially modulate the expression of endogenous *KRAS* as well as its effectors^46,48,64^. This is a particularly important consideration since the functional consequences of oncogenic RAS proteins are highly dependent on their expression; too little RAS signaling is non-transforming, while chronic, moderate levels of RAS are highly transforming, and hyperactive RAS signaling can lead to cellular senescence or growth arrest^34,49,64,65^. This is suggestive of a “Goldilocks Model of Oncogenicity” in which KRAS variants, such as KRAS^G12R^ or KRAS^G13R^, conduct either too little or too much RAS signaling in human lung epithelial cells to effectively drive sustained tumorigenesis. Interestingly, while this model may have predicted that p53 deficiency would enable some otherwise “too strong” Kras variants to drive tumor formation, this was in fact not the case in our study. Finally, the identification of increasing numbers of cancer susceptibility loci using both in vivo and in silico approaches indicate that epistatic interactions between *KRAS* and genetic modifiers represent another intriguing contributor to KRAS mutant oncogenicity^61,66^.

The key insights of our study are twofold: (1) amino acid substitutions in Kras residues 12 and 13 have quantitatively different and cancer type-dependent abilities to drive tumorigenesis and (2) both the functional consequences of specific *Kras* mutations, as well as the mechanisms leading to their initiation, play major roles in determining their pervasiveness in human cancer. Multiplexed approaches that enable the genetic dissection of RAS function in vivo represent a valuable complement to ongoing computational, biochemical, and cell culture-based studies of mutant forms of RAS proteins^6^. Notably, despite mounting evidence that cancers harboring different *KRAS* mutations can have differential therapeutic responses, the singular genetic inclusion criterion of “confirmed *KRAS* mutation” is listed for the vast majority of clinical trials in patients with KRAS-driven tumors.

The development and application of novel strategies to more rapidly interrogate the functional consequences of defined cancer mutations will undoubtedly redefine our biological and clinical understanding of many cancers types. We envision that this CRISPR-based somatic HDR platform will enable an unprecedented understanding of the function of common and rare mutations in oncogenes across diverse cancer types, and will dramatically accelerate both the discovery and pre-clinical validation of therapies for precisely defined genetic subtypes of cancer.

## Methods

### Design, generation, and screening of sgRNAs targeting *Kras*

To obtain an sgRNA targeting *Kras* to enhance HDR in somatic mouse cells, we identified all possible 20 bp sgRNAs (using the consensus Cas9 PAM: NGG) targeting *Kras* exon 2 and the flanking intronic sequences, and scored them for predicted on-target cutting efficiency using an available sgRNA design/scoring algorithm^67^. We then empirically determined the cutting efficiency of three sgRNAs targeting *Kras* (sg*Kras*#1: GCAGCGTTACCTCTATCGTA; sg*Kras*#2: GCTAATTCAGAATCACTTTG; sg*Kras*#3: GACTGAGTATAAACTTGTGG) (Supplementary Fig. 1a). Briefly, Lenti-U6-sgRNA/*Cre* vectors were generated for each sgRNA targeting *Kras* as previously described^4^. Q5^®^ site-directed mutagenesis (NEB) was used to insert the sgRNAs into a parental lentiviral vector containing a U6 promoter to drive sgRNA transcription as well as a PGK promoter to drive *Cre*-recombinase. The cutting efficiency of each sg*Kras* was determined via transduction of *LSL-YFP;Cas9* cells in culture with each Lenti-sg*Kras*/*Cre* virus. We isolated YFP^positive^ cells 48 h post transduction by fluorescence-activated cell sorting (FACS), extracted DNA, PCR-amplified the targeted *Kras* locus (forward primer: TCCCCTCTTGGTGCCTGTGTG; reverse primer: AAGCCCTTCCTGCTAATCTCGGAG), and Sanger-sequenced the amplicons (sequencing primer: GCACGGATGGCATCTTGGACC). Sequencing traces were analyzed by TIDE (tracking of indels by decomposition) to determine percent indel induction^68^. Since all three sgRNAs induced indels at the anticipated loci, the sg*Kras* targeting the sequence closest to *Kras* codons 12 and 13 (sg*Kras*#3) was used for all subsequent experiments as this was expected to best facilitate HDR at the desired locus (Supplementary Fig. 1a).

### AAV-*Kras*^*HDR*^ library design, construction, and validation

The U6-sg*Kras*/PGK-*Cre* cassette from pLL3.3;U6-sg*Kras*/PGK-*Cre* was PCR-amplified with Q5^®^ polymerase (NEB), TOPO-cloned (Invitrogen), and verified by sequencing. To generate the AAV-sg*Kras*/*Cre* vector backbone, the sequence between the inverted terminal repeats (ITRs) of a modified AAV2 transfer vector backbone plasmid was removed using *Xho*I/*Spe*I^69^. Then, the U6-sg*Kras*/PGK-*Cre* cassette was digested from the TOPO vector with *Xho*I/*Xba*I and the 1.9-kb fragment was ligated into the *Xho*I/*Spe*I-digested AAV2 backbone, destroying the *SpeI* site. A BGH polyA sequence was inserted 3′ of *Cre* via *Mlu*I digestion. Lastly, a 2-kb homology arm was generated from the region surrounding exon 2 of murine *Kras* via PCR amplification (forward primer: GCCGCCATGGCAGTTCTTTTGTATCCATTTGTCTCTTTATCTGC; reverse primer: GCCGCTCGAGCTCTTGTGTGTATGAAGACAGTGACACTG). The amplicon was subsequently cloned into a TOPO vector (Invitrogen). *Avr*II/*Bsi*WI sites were introduced into the TOPO-cloned 2-kb *Kras* fragment using Q5^®^ site-directed mutagenesis (NEB) (*Avr*II forward primer: TGAGTGTTAAAATATTGATAAAGTTTTTG; *Avr*II reverse primer: CCTagGTGTGTAAAACTCTAAGATATTCC; *Bsi*WI forward primer: CTTGTAAAGGACGGCAGCC; *Bsi*WI reverse primer: CGtACGCAGACTGTAGAGCAGC; restriction sites are underlined with mismatching bases in lowercase). The *Kras* fragment harboring *Avr*II/*Bsi*WI sites was released from TOPO with *Nco*I/*Xho*I and ligated into *Nco*I/*Xho*I-digested AAV-sg*Kras*/*Cre* to produce the final AAV-*Kras* ^*HDR*^/sg*Kras*/*Cre* backbone vector.

To generate the control AAV-*Kras* ^*HDR*^ backbone without the sgRNA targeting *Kras*, PGK-*Cre* was excised from a TOPO clone with *Not*I/*Xba*I and ligated into the *Not*I/*Xba*I-digested modified AAV2 plasmid backbone. A BGH polyA sequence and the mouse *Kras* homology arm were added as described above to produce the control AAV*-Kras* ^*HDR*^/*Cre* backbone vector.

To introduce a library of activating single point mutations and a DNA barcode into the *Kras* ^*HDR*^ sequence of the AAV backbones, we synthesized four separate 295 bp *Kras* fragments with a degenerate “N” base (A, T, C, or G) at each of the first two basepairs of *Kras* codons 12 or13 (Integrated DNA Technologies) (Supplementary Fig. 1b). By design, each of the four fragment pools consisted of three non-synonymous, single nucleotide mutations at codons 12 and 13 as well as the wild-type *Kras* sequence to serve as a control. Thus, since each of the four pools contained wild-type fragments, the overall representation of wild-type *Kras* alleles was expected to be approximately four times higher than each of the mutant *Kras* alleles. The synthesized fragments also contained silent (synonymous) mutations within the sg*Kras* target sequence and the associated PAM* to render the HDR template resistant to Cas9-mediated editing. Additionally, an eight-nucleotide random barcode was engineered into each fragment by introducing degenerate bases into the wobble positions of the downstream *Kras* codons to enable individual tumor barcoding (Supplementary Fig. 1b). Finally, each fragment included flanking *Avr*II and *Bsi*WI restriction sites for cloning into the AAV-*Kras* ^*HDR*^ backbones (Supplementary Fig. 1b).

The four synthesized fragment pools were combined at equal molar ratios and PCR-amplified (forward primer: CACACCTAGGTGAGTGTTAAAATATTG; reverse primer: GTAGCTCACTAGTGGTCGCC). Amplicons were digested with *Avr*II/*Bsi*WI, purified by ethanol precipitation, and ligated into both AAV-*Kras* ^*HDR*^ backbones (Supplementary Fig. 1c). Each ligated plasmid library was transformed into Stbl3 electro-competent cells (NEB) and plated onto 20 × 10 cm LB-Amp plates, which generated ~3 × 10^5^ bacterial colonies per library. Colonies were scraped into LB-Amp liquid media and expanded for 6 h at 37 °C to increase plasmid yields to obtain enough plasmid DNA for AAV production. Plasmid DNA was then extracted from bacterial cultures using an endotoxin-free Maxiprep kit (Qiagen).

To determine the representation of each *Kras* variant and the distribution of barcode nucleotides within each AAV plasmid library, purified AAV plasmid libraries were PCR-amplified with primers tailed with Illumina^®^ adapters (lowercase) containing multiplexing tags (underlined N′s) (forward primer: aatgatacggcgaccaccgagatctacactctttccctacacgacgctcttccgatctCTGCTGAAAATGACTGAGTATAAACTAGTAGTC; reverse primer: caagcagaagacggcatacgagatNNNNNNgtgactggagttcagacgtgtgctcttccgatcCTGCCGTCCTTTACAAGCGTACG**)**, and then deep-sequenced on a MiSeq sequencer (Illumina^®^). The universal bases between *Kras* codon 12 and the barcode region were used to filter proper MiSeq reads. The specific *Kras* codon 12 or 13 sequence and random barcode associated with each read was then cataloged to determine the representation of the *Kras* variants and the distribution of barcode nucleotides within each AAV plasmid library.

### Availability of AAV-*Kras*^*HDR*^/sg*Kras*/*Cre* vectors

A uniquely barcoded AAV-*Kras* ^*HDR*^/sg*Kras*/*Cre* plasmid vector for each of the 13 *Kras* ^*HDR*^ alleles is available from Addgene (#99848-99860).

### Analysis of lung transduction by AAV capsid serotypes

Recombinant ssAAV-GFP vectors were produced using a Ca_3_(PO_4_)_2_ transient triple transfection protocol with pAd5 helper, pAAV-RSV-GFP transfer vector, and pseudotyping plasmids for each of nine capsids of interest: AAV1, 2, 3b, 4, 5, 6, 8, 9_hu14, and DJ. Vector lots were produced in HEK293T cells (ATCC) followed by double cesium chloride density gradient purification and dialysis as previously described^70^. Recombinant AAV vector preparations were titered by TaqMan quantitative PCR (qPCR) for GFP (forward primer: GACGTAAACGGCCACAAGTT; reverse primer: GAACTTCAGGGTCAGCTTGC; probe: 6-FAM/CGAGGGCGATGCCACCTACG/BHQ-1). To identify an optimal AAV serotype for adult lung epithelial cell transduction, each mouse received 60 µL of pseudotyped AAV-GFP at maximal titer via intratracheal administration (Supplementary Fig. 2). Mice were analyzed 5 days after AAV administration. Lungs were dissociated into single-cell suspensions and prepared for FACS analysis of GFP^positive^ cells as described previously^71^. GFP^positive^ percentages were determined by analyzing >10,000 viable (DAPI^negative^) lung epithelial (CD45/Ter119/F4-80/CD31^negative^, EpCAM^positive^) cells.

### Production and titering of AAV-*Kras*^*HDR*^ plasmid libraries

AAV libraries were produced using a Ca_3_(PO_4_)_2_ triple transfection protocol with pAd5 helper, pAAV2/8 packaging plasmid, and the barcoded *Kras* library transfer vector pools described above. Transfections were performed in HEK293T cells (ATCC) followed by double cesium chloride density gradient purification and dialysis as previously described^70^. AAV libraries were titered by TaqMan qPCR for *Cre* (forward primer: TTTGTTGCCCTTTATTGCAG; reverse primer: CCCTTGCGGTATTCTTTGTT; probe: 6-FAM/TGCAGTTGTTGGCTCCAACAC/BHQ-1).

### In vitro AAV/Cas9-mediated HDR

The nucleotide changes surrounding the mutations at codon 12 and 13 (three nucleotide changes 5′ of codons 12/13 to mutate the sgRNA recognition site and PAM motif, and up to 11 changes in the barcode sequence) made it unlikely that the point mutations at *Kras* codons 12 and 13 would differentially affect the rate of HDR. We nevertheless tested whether HDR efficiency might be influenced by differences in the sequence of individual *Kras* ^*HDR*^ alleles. To induce in vitro AAV/Cas9-mediated HDR, we transduced *LSL-YFP;Cas9* cells with the purified AAV-*Kras* ^*HDR*^/sg*Kras*/*Cre* library (Fig. 2d). Cells were maintained in cell culture media with 10 µM SCR7 (Xcessbio), an inhibitor of non-homologous end joining (NHEJ), to promote HDR. Ninety-six hours after transduction, genomic DNA was isolated from the *LSL-YFP;Cas9* cells by phenol/chloroform extraction followed by ethanol precipitation. The *Kras* locus was amplified using a PCR strategy we developed for the specific amplification of *Kras* ^*HDR*^ alleles integrated into the endogenous *Kras* locus (Fig. 2e). We then deep-sequenced these amplicons to determine the representation of *Kras* ^*HDR*^ alleles following in vitro HDR (see the “Illumina^®^ library preparation and sequencing of tumor barcodes from bulk tissue” section below for details on PCR and sequencing; Fig. 2d–f).

### Tumor initiation in mice using AAV/Cas9-mediated HDR

*Lkb1* ^*flox*^(*L*), *p53* ^*flox*^ (*P*), *R26* ^*LSL-tdTomato*^ (*T*), *H11* ^*LSL-Cas9*^, and *Kras* ^*LSL-G12D*^(*K*) mice have been previously described^9,19–22^. AAV administration by intratracheal inhalation to initiate lung tumors (Fig. 3a and Supplementary Fig. 3a), retrograde pancreatic ductal injection to initiate pancreatic tumors (Fig. 4a and Supplementary Fig. 7a), and intramuscular gastrocnemius injection to initiate sarcomas (Fig. 5a and Supplementary Fig. 8a) were performed as described^19,43,72^. Lung tumors were initiated in *PT*;*H11* ^*LSL-Cas9*^, *LT*;*H11* ^*LSL-Cas9*^, and *T*;*H11* ^*LSL-Cas9*^ mice with 60 µL of AAV-*Kras* ^*HDR*^/sg*Kras*/*Cre* (1.4 × 10^12^ vector genomes/mL (vg/mL)), in *PT*, *LT*, and *T* mice with 60 µL of AAV-*Kras* ^*HDR*^/*Cre* (2.4 × 10^12^ vg/mL), or in *KPT* and *KLT* mice with 60 µL AAV-*Kras* ^*HDR*^/sg*Kras*/*Cre* (1.4 × 10^12^ vg/mL) diluted 1:10,000 in 1× PBS. Pancreatic tumors were initiated in *PT*;*H11* ^*LSL-Cas9*^ mice with 100–150 µL of AAV-*Kras* ^*HDR*^/sg*Kras*/*Cre* (1.4 × 10^12^ vg/mL) or in *PT* mice with 100–150 µL of AAV-*Kras* ^*HDR*^/*Cre* (2.4 × 10^12^ vg/mL). A 1:10 dilution of AAV-*Kras* ^*HDR*^/sg*Kras*/*Cre* in 1× PBS was also administered to the lungs or pancreata of mice where indicated. Sarcomas were initiated in the gastrocnemii of *PT*;*H11* ^*LSL-Cas9*^ mice with 30 µL of AAV-*Kras* ^*HDR*^/sg*Kras*/*Cre* (5.2 × 10^12^ vg/mL). Sample sizes were chosen based on previous studies from our group and others using virally induced mouse models of cancer, and accounting for the anticipated rate of AAV/Cas9-mediated HDR. Male and female mice were distributed as evenly as possible across experimental cohorts, though littermates were otherwise randomly assigned. Following viral transduction, mice were analyzed when they displayed symptoms of tumor development. Within practical limitations, investigators were blinded to mouse cohort assignments when performing all downstream analyzes up to sequencing data analysis. The Institutional Animal Care and Use Committee of Stanford University approved all mouse procedures.

### Analysis of individual lung tumors

Lung tumor-bearing mice displaying symptoms of tumor development were analyzed 4–10 months after viral administration. Lung tumor burden was assessed by lung weight and quantification of macroscopic Tomato^positive^ tumors under a fluorescence dissecting scope as indicated (Fig. 3b, c and Supplementary Fig. 3b–e) (a single *LT*;*H11* ^*LSL-Cas9*^ mouse had minimal Tomato^positive^ signal that was restricted to a small region of one lung lobe, indicative of improper intratracheal administration of AAV, and was therefore removed from the study). The largest individual lung tumors that were not visibly multifocal were dissected from bulk lungs under a fluorescence dissecting microscope for sequencing. For the majority of lung tumors, Tomato^positive^ (and DAPI/CD45/CD31/F4-80/Ter119^negative^) cancer cells were purified using FACS instruments (Aria; BD Biosciences) within the Stanford Shared FACS Facility. Several individually dissected tumors were not purified by FACS. Lung lobes from mice of each genotype were also collected for histological analyses.

### Analysis of individual pancreatic tumor masses

Pancreatic tumor-bearing mice displayed symptoms of tumor development and were analyzed 3–4 months after viral administration. Since pancreatic tumors generally appeared to be multifocal (Supplementary Fig. 7b), individual regions of the pancreas containing Tomato^positive^ tumor masses were dissected and FACS-purified for sequencing (a mouse treated with a 1:10 dilution of AAV-*Kras* ^*HDR*^/sg*Kras*/*Cre* library also developed pancreatic tumor masses and therefore was included in these analyses). Regions of several pancreata were kept for histological analyses (Fig. 4b, c and Supplementary Fig. 7c).

### Analysis of individual sarcomas

Sarcoma-bearing mice with obvious tumor development were analyzed 3–7 months after viral administration (Fig. 5 and Supplementary Fig. 8). A region of each sarcoma was sequenced and an adjacent region was saved for histological analysis (Fig. 5b, c and Supplementary Fig. 8c).

### Characterization of *Kras* alleles in individual tumors

Genomic DNA for sequencing was extracted from FACS-purified tumor cells and unsorted tumor samples with a DNeasy Blood and Tissue Extraction kit (Qiagen). To identify *Kras* point mutations and tumor barcodes, we PCR-amplified and sequenced the *Kras* ^*HDR*^ alleles using two protocols optimized for several variables including annealing temperature, extension time, and primer sequences: (1) forward primer: CTGCTGAAAATGACTGAGTATAAACTAGTAGTC, reverse primer: AGCAGTTGGCCTTTAATTGGTT, sequencing primer: AATGATACGGCGACCACCGAGATCTACAC, annealing temperature: 66 °C, extension time: 2–3 min; and (2) forward primer: GCTGAAAATGACTGAGTATAAACTAGTAGTC, reverse primer: TTAGCAGTTGGCCTTTAATTGG, sequencing primer: GCACGGATGGCATCTTGGACC, annealing temperature: 64 °C, extension time: 2–3 min. These protocols were used to specifically amplify integrated *Kras* ^*HDR*^ alleles from individual tumors as each incorporated a forward primer overlapping the engineered mutations in the PAM region upstream of codons 12 and 13, and a reverse primer outside the homology arm. Long extension times (2–3 min) were used to enable amplification of all *Kras* ^*HDR*^ alleles, even those containing insertions or duplications in intron 2 of the *Kras* locus (Supplementary Fig. 5d).

A variety of HDR-induced oncogenic *Kras* alleles were identified in 49 individual lung tumors (Fig. 3h), 4 individual pancreatic tumor masses (Fig. 4e), and 5 sarcomas (Fig. 5e). For the *Kras* ^*HDR*^ alleles identified, *p* values for likelihood of enrichment relative to WT (taking into account the initial representation of each *Kras* ^*HDR*^ alleles in the AAV-*Kras* ^*HDR*^/sg*Kras*/*Cre* plasmid library) were generated using a Fisher’s exact test, generalized for structural zeros when no tumors with a WT *Kras* ^*HDR*^ alleles were identified (Fig. 3h)^73^. All *p* values were Bonferroni-corrected for the number of variants investigated and were two-sided.

Apart from introducing the desired point mutations into the endogenous *Kras* locus via HDR, targeting *Kras* exon 2 using CRISPR/Cas9 was also expected to result in indels at the cut site following DNA repair by NHEJ instead of HDR. To characterize these modifications, we used a generic PCR protocol to amplify both *Kras* alleles (forward primer: TCCCCTCTTGGTGCCTGTGTG; reverse primer: GGCTGGCTGCCGTCCTTTAC; sequencing primer: CAAGCTCATGCGGGTGTGTC; annealing temperature: 72 °C). A spectrum of insertions and deletions at the expected target site in the endogenous *Kras* locus was identified in 46 lung tumors using this approach (Supplementary Fig. 6).

For some individual tumor samples, the sequence of both *Kras* alleles was not immediately obvious following the above PCR and sequencing strategies. PCR products from these complicated samples were TOPO-cloned (Invitrogen) and transformed, and several colonies from each sample were plasmid-prepped and sequenced to characterize both *Kras* alleles in each tumor. This approach was reproducible and reliable across both biological and technical replicates.

These analyses uncovered several unexpected features in some *Kras* alleles from individual lung tumors. Three distinct missense mutations at codon 24 (I24L, I24N, I24M) were observed in a small subset of the individual lung tumors analyzed. Further analyses indicated that these mutations were likely SNPs present in some of the genetically engineered mice. The function of these alterations, if any, is unknown.

Furthermore, we initially anticipated that recombination of the *Kras* ^*HDR*^ template into the endogenous *Kras* locus would almost exclusively occur outside the *Avr*II and *Bsi*WI sites engineered into the *Kras* ^*HDR*^ template (Supplementary Fig. 1d). However, the *Avr*II site, engineered by altering 2 base pairs 97 bp upstream of exon 2, was absent in 5 out of 25 tumors in which we directly analyzed this region of the *Kras* ^*HDR*^ allele (Supplementary Fig. 5c). The *Bsi*WI site, engineered by altering 1 base pair 20 bp downstream of exon 2, was absent in 11 out of 58 tumors (Supplementary Fig. 5c). These findings indicated that while recombination of the *Kras* ^*HDR*^ template most often occurred within the larger, more distal homology arms, it also occurred at a detectable frequency within very short regions of homology flanked by 5′ and 3′ mismatches (including the PAM* mutations, a *Kras* codon 12 or 13 mutation, and up to 11 mismatches within the barcode region).

After we initially identified the presence of duplications in *Kras* ^*HDR*^ alleles in some tumors (Supplementary Fig. 5d), we designed PCR primers to specifically amplify duplications of *Kras* exon 2 that occurred on either side of exon 2 within *Kras* ^*HDR*^ alleles (right-hand duplication—forward primer: TGACCCTACGATAGAGGTAACG; reverse primer: CTCATCCACAAAGTGATTCTGA; sequencing primer: TGACCCTACGATAGAGGTAACG; left-hand duplication—forward primer: TGAGTGTTAAAATATTGATAAAGTTTTTG; reverse primer: TCCGAATTCAGTGACTACAGATG; sequencing primer: TGAGTGTTAAAATATTGATAAAGTTTTTG). Each of these duplication-specific PCR protocols used adjacent primer pairs in opposite orientations, ensuring that amplification would only occur if a duplication were present. Duplications of varying lengths were identified, including duplications of the second half of wild-type exon 2 or the entire exon 2 (but lacking critical regions of the splice acceptor) (Supplementary Fig. 5d). Deletions and duplications of regions of intron 2 were also observed. Furthermore, we observed integrations of parts of the AAV transfer vector, including the U6 promoter and viral ITR, into intron 2. Given the size and location of these alterations, none would be expected to change splicing of *Kras* mutant exon 2 to exon 3, consistent with the requirement of oncogenic *Kras* to drive tumorigenesis.

### Generating a normalization control for Tuba-seq

To obtain a normalization control for tumor barcode sequencing, a single large tumor was dissected from an *PT*;*H11* ^*LSL-Cas9*^ mouse, digested into a single cell suspension, and plated to generate a cell line. After expanding these cells and then extracting genomic DNA, *Kras* exon 2 was PCR-amplified (forward primer: TCCCCTCTTGGTGCCTGTGTG; reverse primer: GGCTGGCTGCCGTCCTTTAC). The PCR product was sequenced (using specific and generic sequencing primers described above) to confirm the presence of a *Kras* ^*HDR*^ allele and a barcode. A single *Kras* ^*G12V*^ allele (G**T**T) with a unique barcode (**C**GG**G**AA**G**TC**G**GC**G**CT**T**AC**G**AT**C**) was identified. Genomic DNA from this cell line was used as a normalization control for high-throughput sequencing for all bulk lung samples (Supplementary Fig. 9b).

### Bulk lung tissue processing and DNA extraction

Bulk lung samples were dissected from virally-transduced mice and stored at −80 °C prior to processing. To extract genomic DNA for sequencing, samples were thawed and transferred to 50 mL conical tubes. 20 mL of lysis buffer (100 mM NaCl, 20 mM Tris pH 7.6, 10 mM EDTA pH 8.0, 0.5% SDS in H_2_O) plus 200 µL proteinase K (20 mg/mL) were added to each sample. Next, 3 µg (~5 × 10^5^ genomes) of normalization control DNA was added to each sample (Fig. 6a and Supplementary Fig. 9b). Samples were then carefully homogenized using a tissue blender, which was cleaned between each sample by progressing through clean 10% bleach, 70% ethanol, and 1× PBS. Homogenized samples were lysed at 55 °C overnight. Genomic DNA was isolated from tissue lysates by phenol/chloroform extraction followed by ethanol precipitation.

### Bulk pancreatic tissue processing and DNA extraction

Pancreatic tumor masses were dissected and digested, and viable neoplastic cells (Tomato^positive^, DAPI/CD45/CD31/Ter119/F4-80^negative^) were isolated by FACS. No normalization control was added to the pancreatic cancer samples. Genomic DNA was isolated from FACS-isolated neoplastic cells using a DNeasy Blood and Tissue Extraction kit (Qiagen), and then further purified by ethanol precipitation.

### Illumina^®^ library preparation and Tuba-seq

To uncover the number and size of tumors harboring each *Kras* variant in a massively parallel and quantitative manner, we developed a two-round PCR strategy that enabled multiplexed Illumina^®^ sequencing of barcoded *Kras* ^*HDR*^ alleles (Fig. 2e). For the first round of PCR, we used a forward primer complementary to the *Kras* ^*HDR*^ sequence containing the three PAM and sgRNA target site mutations (PAM*; bold in the first round forward primer sequence) (first round forward primer: GCTGAAAATGACTGAGTATAAACT**A**GT**A**GT**C**), and a reverse primer complementary to a downstream region of the endogenous *Kras* locus not present in the HDR template of the AAV-*Kras* ^*HDR*^/sg*Kras*/*Cre* vector (first round reverse primer: TTAGCAGTTGGCCTTTAATTGG). This primer pair was chosen to specifically amplify genomic *Kras* ^*HDR*^ alleles without amplifying the abundant wild-type *Kras* alleles or potential episomal AAV-*Kras* ^*HDR*^/sg*Kras*/*Cre* vectors present in DNA purified from bulk tumor-bearing tissue. Additionally, a P5 adapter (italicized), an 8 bp custom i5 index (N′s), and an Illumina^®^ sequencing primer sequence (read 1) (underlined) were included at the 5′ end of the first round forward primer to enable multiplexed Illumina^®^ sequencing (first round forward primer for Illumina^®^ sequencing: *AATGATACGGCGACCACCGAGATCTACAC*NNNNNNNNACACTCTTTCCCTACACGACGCTCTTCCGATCTGCTGAAAATGACTGAGTATAAACT**A**GT**A**GT**C**).

Importantly, since the characterization of *Kras* ^*HDR*^ alleles in individual tumors uncovered some variability in HDR (e.g., diverse indels in *Kras* intron 2; Supplementary Fig. 5), only four cycles (for lung samples) or six cycles (for pancreas samples) were performed in the first round of PCR to minimize the potential for bias during the amplification of products of variable length. Furthermore, a high-efficiency polymerase (Q5^®^ hot start high-fidelity polymerase, NEB; 64 °C annealing temperature) and a long extension time (3:00 min) were used to ensure robust amplification of all *Kras* ^*HDR*^ alleles. *Kras* ^*HDR*^ alleles in genomic lung DNA were amplified using between 4 and 40 separate 100-µL PCR reactions and then pooled following amplification to reduce the effects of PCR jackpotting (Supplementary Fig. 9a). Each of these 100-µL PCR reactions contained 4 µg of DNA template to amplify from a large initial pool of *Kras* ^*HDR*^ alleles. Following the first round of amplification, all replicate PCR reactions were pooled and 100 µL of each sample was cleaned up using a QIAquick PCR purification Kit (Qiagen). Purified first round PCR amplicons were used as template DNA for a 100 µL second round Illumina^®^ library PCR (Q5^®^ hot start high-fidelity polymerase, NEB; 72 °C annealing temperature; 35 cycles for lung samples, 40 cycles for pancreas samples). The second round of PCR amplified a 112 bp region entirely within the *Kras* exon 2 sequence present in first round PCR amplicons. The second round reverse primer contained a P7 adapter (italicized), a reverse complemented 8 bp custom i7 index (“Ns”), and a reverse complemented Illumina^®^ sequencing primer sequence (read 2) (underlined) at the 5′ end to enable dual-indexed, paired-end sequencing of Illumina^®^ libraries (second round reverse primer #1: *CAAGCAGAAGACGGCATACGAGAT*NNNNNNNNGTGACTGGACTTCAGACGTGTGCTCTTCCGATCCGTAGGGTCATACTCATCCACA). The second round PCR forward primer was complementary to the P5 Illumina^®^ adapter added to the amplified *Kras* ^*HDR*^ allele by the forward primer during the first round PCR (second round forward primer: AATGATACGGCGACCACCGAGATCTACAC). This primer was used to amplify first round PCR amplicons without amplifying any contaminating genomic DNA that may have been carried over from the first round PCR reaction. Furthermore, a second reverse primer encoding the P7 adaptor sequence was added to the second round PCR reaction at the same concentration as the two other primers (second round reverse primer #2: CAAGCAGAAGACGGCATACGAGAT). This primer binds the reverse complemented P7 adaptor sequence added to the *Kras* ^*HDR*^ amplicons by second round reverse primer #1. Since the second round PCR was performed over 35–40 cycles, the P7 adaptor (second round reverse primer #2) was added to limit the amount of non-specific amplification produced by the lengthy second round reverse primer #1.

After the second round of amplification, 100-µL PCR reactions were run on a 2.5% agarose gel and a band of the expected size was excised. PCR amplicons were extracted from gel fragments using a QIAquick gel extraction Kit (Qiagen). The size, concentration, and purity (i.e., the lack of unexpectedly sized peaks indicative of nucleic acid or buffer contaminants) of the purified Illumina^®^ libraries was confirmed using a Bioanalyzer (Agilent). Individual Illumina^®^ libraries with unique dual-indices were then pooled together such that libraries originally derived from mice with greater tumor burden were represented at a higher ratio in the final pool than those from mice with lower tumor burdens (Supplementary Fig. 9a). A total of 35 individual samples were combined into two Illumina^®^ library pools. The size, concentration, and purity of each pool was again confirmed on a Bioanalyzer (Agilent). Each final Illumina^®^ library pool was then deep-sequenced on an Illumina^®^ HiSeq lane using a multiplexed, 150 bp paired-end Rapid Run sequencing program (Elim Biopharmaceuticals).

### Estimating the size and number of barcoded tumors

We developed a publically available pipeline to call tumors from our de-multiplexed Illumina^®^ sequencing data, termed Tuba-seq (https://github.com/petrov-lab/tuba-seq). The pipeline tallies unique barcode sequences and eliminates recurrent sequencing errors using an algorithm designed to denoise deep-sequencing data of amplicons (*DADA2*)^74^. We tailored this algorithm to minimize the occurrence of spurious tumor calls, and minimize technical biases (including variation in read depth, variation in Illumina^®^ sequencing machine error rates, and variation in barcode diversity). This pipeline was modified here for the analysis of tumor genotypes and barcodes following AAV/Cas9-mediated somatic HDR-driven tumorigenesis, as described below.

Although our Illumina^®^ sequencing libraries contained a small 112 bp fragment of the *Kras* ^*HDR*^ alleles, we performed 150 bp paired-end sequencing of these fragments and merged the overlapping forward and reverse reads to reduce the likelihood of Illumina^®^ sequencing errors in *Kras* codons 12 and 13 and the barcode region of the *Kras* ^*HDR*^ alleles. Overlapping paired end-reads were merged, quality-filtered, and trimmed using PANDAseq (fragment length: 60 bp; forward trimming primer: ATGACTGAGTATAAACT; reverse trimming primer: CTCATCCACAAAGTGA)^75^.

Even after merging forward and reverse reads to reduce sequencing errors, an average of ~1 error per 10,000 bases was detected, presumably from recurrent Illumina^®^ sequencing errors or from errors introduced during PCR. Given this error rate, we expected that reads from a large, uniquely barcoded tumor containing single nucleotide mismatches would be called as small, spurious tumors of ~1/10,000th the size of the large real tumor. Even without Tuba-seq, this phenomenon was discernible by eye, as we observed small clusters of spurious “tumors” that were ~3–4 orders of magnitude smaller and contained 1 nucleotide deviation relative to the largest tumor in specific mice. Additionally, each *Kras* ^*HDR*^ variant-barcode pair also possessed recurrent sequencing errors in mutant base in oncogenic *Kras* codon 12 or 13.

To accurately call tumors, we developed a computational and statistical pipeline for the analysis of tumor barcode sequencing data. We first estimated the residual rate of sequencing/PCR errors from the seven nucleotides upstream of *Kras* codon 12 and the seven nucleotides downstream of the final barcode base. We then used our model of sequencing errors to cluster unique read pileups (truncated to within seven nucleotides of the barcoded bases) into unique tumors via *DADA2*. A minimum confidence in unique origin of the clusters of 0.01 (i.e., omega_a = 0.01) was used. A larger threshold increased the number of unique tumors called in a mouse sample. We chose this larger value rather than those used previously^26^, as paired-end sequencing gave us greater confidence that unique read pileups were truly distinct tumors. For example, we found that this threshold eliminated all unintended read sequences (e.g., reads with inappropriate nucleotides outside of the barcode), and that this threshold called a total number of lesions within each mouse more consistently between biological replicates. These were important considerations since without proper handling of read errors, the number of called tumors can positively correlate with sequencing read depth. Finally, we removed any tumors with DNA sequences that deviated by only 1 nucleotide from a lesion that was 10,000× larger. This affected only 1.56% of tumor calls.

After generating the read pileups and performing the corrections described above, we normalized the number of reads from each called tumor to the number of reads from the normalization control that was spiked into each sample prior to DNA extraction from bulk tumor-bearing tissue lysates (Fig. 6a and Supplementary Fig. 9b). This allowed us to generate a reasonable estimate of the number of neoplastic cells in each tumor and allowed us to merge data from mice of the same genotype and treatment. However, there are several factors that impact our ability to accurately quantify the absolute number of neoplastic cells within each tumor.

First, some *Kras* ^*HDR*^ alleles in individual tumors harbored insertions or deletions in *Kras* intron 2, inside the first round PCR primers for Illumina^®^ sequencing. Although the presence of different sized amplicons could generate a PCR bias, we attempted to reduce this by performing only four to six cycles in the first round of Illumina^®^ library PCR, using a long extension time (~3 min), and using a fast (20–30 s/kb), high fidelity polymerase (Q5^®^; NEB). As the final Illumina^®^ library PCR product in the second round of amplification is short and uniform across all samples, PCR amplification should not be biased at this step.

Second, given that the *Kras* variants and barcodes are knocked into the endogenous *Kras* locus, it is possible that in some tumors this region is genomically amplified (which has been documented in *Kras* ^*G12D*^-driven lung tumors initiated in mouse models of lung cancer^31,76^). Although *Kras* amplifications do not typically result in very high *Kras* copy numbers, any amplification of this region would lead to an overestimation of the number of neoplastic cells in tumors with amplified *Kras* ^*HDR*^ alleles since our conversion from read count to cell number assumes that each cell contains a single copy of the barcoded *Kras* ^*HDR*^ allele.

Lastly, the normalization control itself was generated from cells from a tumor with a known duplication in *Kras* intron 2, which produces a larger PCR product in the first round of the Illumina^®^ library preparation than tumors without a duplication. Thus, any PCR bias away from the *Kras* ^*HDR*^ allele in the normalization control would result in a systematic overestimation of the size of tumors.

Sequencing of tumor barcodes from 35 samples on two lanes of an Illumina^®^ Hi-Seq Rapid Run, combined with our analysis pipeline, enabled the detection of unique barcodes with read counts covering five orders of magnitude. Thus, the resolution of this approach enables detection of both large lesions and small hyperplasias within bulk tissue. However, the ability to detect a large numbers of lesions within bulk tissue increases the probability of barcode collisions: the occurrence of two or more lesions with the same DNA barcode in the same mouse. Barcode collisions can overstate the size of observed tumors because two small “colliding” tumors would be identified as a single, larger tumor. Therefore, we developed a statistical model of barcode collisions to reduce this likelihood and not overtly bias the estimated sizes of called tumors.

Our model of barcode collisions accounts for the likelihood *p*_i_ of observing each of the 24,576 possible barcodes *i* for each *Kras* variant in our study. A majority of the reproducible variation between barcode frequencies in our pool derives from statistically independent variation in the nucleotide frequencies at each wobble base (i.e., each barcode is not equally likely in the pool because there was subtle variation in nucleotide concentrations during synthesis of the barcodes fragments) (Fig. 2c). Thus, we estimate the independent frequency *f*_*b*,*n*_ of each nucleotide n at every base *b* in the barcode and use this data to predict barcode likelihoods based on each barcode’s sequence *B*_*i*,*b*,*n*_ (where *B* is 1 if barcode *i* possesses nucleotide *n* at position *b* and 0 otherwise) as follows:

$${p_i} = \mathop {\prod }\limits_b {B_{i,b,n}} \cdot {f_b}^n$$

Here, superscript indices represent contravariant vectors and the dot represents matrix multiplication. This model predicts every barcode’s frequency with only 21 free parameters. Because some residual over-representation of barcodes persisted in the lung samples, we discarded the 10% most frequently observed barcodes, after correcting for nucleotide frequencies, from all lung analyses. These most frequently observed barcodes were identified independent of our mouse experiments by Illumina^®^ sequencing (MiSeq) of our pAAV-*Kras* ^*HDR*^/sg*Kras*/*Cre* plasmid pool prior to viral production. After this processing, we then renormalized ∑_*i*_ *p*_*i*_ to one.

We then assumed that the occurrences of each barcode within each mouse was a multinomial sampling process. The mean number of collisions *C*_*i*_ for each observed barcode within each mouse is then:

$$\begin{array}{ccccc} {C_i}\left( {{p_i},N} \right)\hskip -8pt &=&\hskip -22pt \mathop {\sum}\limits_{k = 1}^\infty {\left( {k - 1} \right)P\left( {k;{p_i},N} \right)} \\ & =&\hskip -8pt {\mu _i} - P\left( {k = 0;{p_i},N} \right) - 1\\ &=&\hskip -22pt N{p_i} + {\left( {1 - {p_i}} \right)^N} - 1 \end{array}$$

Here, ${\mu _i}$ denotes the mean number of barcodes within each mouse, while *N* denotes the total number of tumors (both unknowns). *N* is then determined from the observed number of tumors in each mouse *N* ^(*obs*)^ using the equation ${N^{(obs)}} = N - \mathop {\sum}\nolimits_i {{C_i}\left( {{p_i},N} \right)}$, which is solved using Brent’s Method.

This model found that barcode collisions were generally rare in our mouse samples (on average 4.04%). However, the likelihood of collisions can vary by mouse and by *Kras* variant. For example, the average predicted number of collisions for WT *Kras* ^*HDR*^ alleles was 5.8% and as high as 12% in one mouse. WT *Kras* ^*HDR*^ alleles were expected to experience the highest number of collisions since WT *Kras* vectors were intentionally represented approximately fourfold more than each mutant *Kras* vector in the initial pAAV-*Kras* ^*HDR*^/sg*Kras*/*Cre* plasmid pool (Fig. 2b). Thus, we divided the size of each lesion by 1 + *C*_i_ to minimize the bias that barcode collisions impart on tumor size distributions. Because collisions are rare events, the particular number of collisions within each mouse can differ substantially from *C*_i_. Because of this limitation, we believe that this correction minimizes systematic bias in tumor size distributions resulting from barcode collisions that would alter our final estimates of the mean effect of each *Kras* variant; however, it cannot effectively identify the specific collisions that occurred among specific barcodes in specific mice.

To determine whether *Kras* variants had quantitatively different abilities to drive tumorigenesis, we elected to focus on lung tumors estimated to contain > 100,000 neoplastic cells (i.e., one-fifth the “size” of the normalization control DNA added to each sample, which was derived from ~5 × 10^5^ cells). Regression analysis of lung tumors above this cell number from replicate samples (independent sample preparation, sequencing, and processing) demonstrated high correlation (all *R* ^2^ values were above 0.99; Supplementary Fig. 10). We believed that the estimated number of cells in tumors below this cutoff was less reliable; *Kras* ^*HDR*^ variant-barcode pairs present at low read counts were much more likely to be biased by barcode collisions and variability in both PCR amplification and DNA sequencing. Additionally, we could not rule out the possibility that these variant-barcode pairs were a product of off-target amplification of our AAV-*Kras* ^*HDR*^/*Cre* control vector, which contains a longer *Kras* ^*HDR*^ template that partially overlaps the reverse primer used for Illumina^®^ library preparation of bulk samples with AAV-*Kras* ^*HDR*^/sg*Kras*/*Cre*-initiated tumors (as opposed to small but real HDR-initiated lung hyperplasias). We anticipate that future technical and computational improvements will further improve the resolution of this pipeline and enable tumor calling at much smaller cell number cutoffs.

Since *Kras* expression can be influenced by overall changes in codon usage that effect tumorigenesis^46,47^, we also determined whether the use of different nucleotides in the barcode region of *Kras* ^*HDR*^ alleles had a detectable effect on lung tumor size. To do this, we calculated the relative change in codon usage generated by the unique barcode in each lung tumors. The barcode region of each *Kras* ^*HDR*^ allele contains three anchor bases that are different from wild-type *Kras* (but universal to all *Kras* ^*HDR*^ alleles), as well as random nucleotides in the eight positions that make up the barcode. A “codon usage score” for the *Kras* ^*HDR*^ allele in each lung tumor was calculated as the sum of the mouse codon frequencies (from a publicly available mouse codon usage table^48^) across the 22 bp barcode region. A codon usage score was also calculated for wild-type *Kras* and for a “minimal *Kras* ^*HDR*^ allele” containing the three universal anchor bases, but wild-type bases at all eight positions of the barcode (Supplementary Fig. 12).

### Analysis of sequencing data from bulk tumor-bearing lungs

We quantified the relative number of tumors harboring each *Kras* variant by counting tumors above 100,000 cells in six *PT*;*H11* ^*LSL-Cas9*^, six *LT*;*H11* ^*LSL-Cas9*^, and three *T*;*H11* ^*LSL-Cas9*^ mice (Supplementary Fig. 9a). Read counts from tumors with each *Kras* variant were normalized by dividing by the initial representation of each variant in the AAV-*Kras* ^*HDR*^/sg*Kras*/*Cre* plasmid pool (for this analysis, the initial representation of each variant in the plasmid pool was calculated from the total number of reads associated with each *Kras* variant after removing barcodes above the 98th percentile of barcode abundance; this restriction did not appreciably alter results, and was simply applied to ensure that extremely abundant variant-barcode pairs did not overtly impact the overall representation of specific variants).

Relative tumor number was then scaled such that WT *Kras* variants had a representation of 1. A small number of WT *Kras* ^*HDR*^ alleles appeared to arise from tumors above 100,000 cells. These could represent tumors in which an HDR event created the non-oncogenic Kras WT genotype but which nonetheless evolved into a tumor for other reasons, or the WT *Kras* variant “hitchhiked” with an oncogenic *Kras* variant by co-incident HDR in the same lung cell followed by expansion driven by the oncogenic variant.

A small number of residual cells from individual tumors dissected from bulk tissue (and analyzed as described above) were usually detectable in our bulk tumor sequencing data. In all analyses of tumor size, these dissected tumors were excluded as we could not infer their true size. However, when analyzing the number of tumors above 100,000 cells in each treated mouse genotype, we included data from individually dissected tumors since dissectible tumors were always among the largest tumors observed within any mouse and, therefore, certainly above the 100,000 cell threshold.

Statistically significant differences in tumor number were determined using Fisher’s exact test. For each variant, two tests were performed, comparing with either the frequency of G12D or WT *Kras* ^*HDR*^ alleles (Fig. 6b–e). All *p* values were Bonferroni-corrected for the number of variants investigated and were two-sided. A two-sided “many cells” Pearson’s *χ* ^2^ test was used to compare the distribution of tumor numbers across all *Kras* variants in *PT*;*H11* ^*LSL-Cas9*^ and *LT*;*H11* ^*LSL-Cas9*^ mice relative to *T*;*H11* ^*LSL-Cas9*^ mice (Supplementary Fig. 11f).

### Analysis of sequencing data from bulk pancreas tumor masses

Following Illumina^®^ sequencing and read processing as described above, we quantified the number of pancreatic tumors with each *Kras* ^*HDR*^ allele in four primary tumor masses and three metastasis samples from two *PT*;*H11* ^*LSL-Cas9*^ mice transduced with AAV-*Kras* ^*HDR*^/sg*Kras*/*Cre* by retrograde pancreatic ductal injection (Supplementary Fig. 13a). Since we did not add normalization control DNA to these samples, we used a stringent internal read count cutoff of two times the most abundant WT *Kras* ^*HDR*^ allele in each sample. Combined with the four tumors identified by individual pancreatic tumor analysis (Fig. 4e), we identified 25 primary tumors containing *Kras* ^*HDR*^ alleles across three *PT*;*H11* ^*LSL-Cas9*^ mice. One *Kras* ^*HDR*^ allele with a specific variant-barcode pair was identified in an individually dissected tumor as well as in the bulk tumor mass from the same mouse and was therefore only counted once. Two *Kras* ^*HDR*^ alleles with specific variant-barcode pairs were discarded since they were thought to be contamination from cell lines generated from individual pancreatic tumors with the same specific variant-barcode pairs. Tumor number counts for each *Kras* ^*HDR*^ allele were then normalized to the initial representation of each allele in the AAV-*Kras* ^*HDR*^/sg*Kras*/*Cre* library. Statistically significant differences in the number of tumors with each *Kras* ^*HDR*^ allele were determined by a Fisher’s exact test for likelihood of enrichment relative to WT (Fig. 7b). All *p* values were Bonferroni-corrected for the number of variants investigated and were two-sided. Spatial and phylogenetic relationships between primary tumors and metastases were established through shared *Kras* ^*HDR*^ variant-barcode pairs (Fig. 7c and Supplementary 13b).

### Comparing the oncogenicity and biochemistry of Kras variants

To identify any relationships between the oncogenicity of Kras mutants observed in vivo and differences in their biochemical properties, we compared our data to the biochemical data for several of the KRAS variants reported in Hunter et al.^2^ (Supplementary Fig. 14)^48^. Specifically, intrinsic and P120GAP-stimulated GTP hydrolysis rates (Hunter et al.^2^; Supplementary Data 1), as well as relative RAF kinase affinities (Hunter et al.^2^; Supplementary Data 2), were obtained for human KRAS^G12D^, KRAS^G12V^, KRAS^G12R^, KRAS^G12C^, KRAS^G12A^, and KRAS^G13D^. Lung and pancreatic tumor numbers plotted against this biochemical data are shown in Supplementary Fig. 14.

### Analysis of KRAS mutations in human cancer genomics data sets

The prevalence of *KRAS* mutations in human lung adenocarcinoma, PDAC, and rhabdomyosarcoma was quantified from human cancer genomics data available in the Catalog of somatic mutations in cancer (COSMIC; release v81) and the American Association for Cancer Research (AACR) Project Genomics Evidence Neoplasia Information Exchange (GENIE; version 1.0.1) databases, as well as from other independent studies, as outlined below (Supplementary Fig. 15 and Supplementary Data 4). COSMIC mutation data from both targeted and genome-wide screens were utilized (http://www.cancer.sanger.ac.uk/cosmic)^77^. The data were obtained, in part, from cBioPortal^78,79^.

To determine the prevalence of *KRAS* mutations in human lung adenocarcinoma, we compiled data from COSMIC and GENIE as well as several additional studies (Supplementary Data 4)^58,78,79^. All samples were screened for duplicates across databases by comparing sample identifier numbers. All remaining samples were pooled to determine both the overall frequencies of somatic mutations in *KRAS* and the spectrum of specific *KRAS* mutations observed in lung adenocarcinoma (Supplementary Fig. 15a and Supplementary Data 4).

We also compared the spectrum of *KRAS* mutations in lung adenocarcinomas in “never-smoker” and “current/former-smoker” patients from various studies (Supplementary Fig. 16b and Supplementary Data 6). Patients whose smoking history was unknown were not included in the analysis. “Never-smokers” included patients that reported smoking fewer than 100 cigarettes in their lifetime as well as patients described as “nonsmokers.” “Current/former-smokers” included patients described as “former” or “current” smokers in the included studies, as well as individuals described as “smokers.”

To determine the prevalence of *KRAS* mutations in human PDAC, we combined data from COSMIC and GENIE, as well as The Cancer Genome Atlas (TCGA) provisional data set (NIH NCI Genomic Data Commons Release 7.0) and one additional study^50^. We screened for duplicate samples by comparing sample identifier numbers. All remaining samples were pooled to estimate the frequency of somatic mutations in *KRAS*, as well as the spectrum of specific *KRAS* mutations in patients with PDAC (Supplementary Fig. 15c and Supplementary Data 4).

To examine *KRAS* mutations in patients with rhabdomyosarcoma, we similarly compiled data from COSMIC and GENIE, as well as from numerous independent studies (Supplementary Data 4). We screened for duplicate samples by comparing sample identifier numbers. Remaining samples were pooled to estimate overall *KRAS* mutation frequency and the specific spectrum of *KRAS* mutations observed in patients with rhabdomyosarcoma (Supplementary Fig. 15b and Supplementary Data 4).

### Approximating relative *KRAS* mutation frequencies

We approximated the relative induction rate of mutations in *KRAS* codons 12 and 13 from data reported in Campbell et al.^58^ Campbell et al.^58^ document six mutational signatures in lung adenocarcinomas: a smoking-related signature (SI4), a molecular clock-like signature (SI5), an ultraviolet (UV)-related signature (SI7), a mismatch-repair signature, and two APOBEC-related signatures (SI13 and SI2). Since we were interested in estimating the relative rate of induction of *KRAS* codons 12 and 13 mutations prior to tumorigenesis, we focused on the mutational processes presumed to be tumor-extrinsic—the smoking-related signature and the molecular clock-like signature. Campbell et al.^58^ (Supplementary Data 7) contains the estimated probabilities of every possible nucleotide substitution within each trinucleotide motif (i.e., the substitution plus the adjacent 5′ and 3′ bases) for each of the mutational signatures. To approximate the relative frequency of specific substitutions in *KRAS* codons 12 and 13, we summed the trinucleotide substitution probabilities associated with the smoking- and molecular clock-like signatures for each of the *KRAS* mutations. Normalizing these probabilities within the 12 single-nucleotide mutations in *KRAS* codon 12 or 13 allowed us to approximate their average relative frequencies in the cells of origin of the lung adenocarcinomas analyzed in Campbell et al. (Supplementary Data 6, current study). These relative frequencies were used to normalize the human data in Supplementary Fig. 16a.

### Comparison of mouse vs. human Kras

Coding and amino acid sequences for mouse Kras (Kras_GRCm38.p4_CCDS20693.1) and human KRAS (Kras_GRCh38.p7_CCDS8702.1) were downloaded from Ensembl (http://www.ensembl.org; Release 89)^80^. Amino acid identity was assessed in a pairwise fashion across each of the 188 amino acids of KRAS. Codon usage for each species was calculated using publicly available codon usage tables^48^. The log-2 fold change in the usage of each codon relative to average (i.e., 1/64) was calculated (Supplementary Fig. 17).

### Data availability

All raw Illumina^®^ sequencing data have been deposited in the NCBI Sequence Read Archive (SRA) with study number SRP119241 (accesion numbers for the raw sequencing files are SRR6124017–SRR6124051). Wherever possible, the processed data used to generate graphs for Figures and Supplementary Figures are also available in numerical form in the Supplementary Data files.

## Electronic supplementary material

Supplementary Information

Description of Additional Supplementary Files

Supplementary Data 1

Supplementary Data 2

Supplementary Data 3

Supplementary Data 4

Supplementary Data 5

Supplementary Data 6
