# Supplementary material for: Multiplexed in vivo homology-directed repair and tumor barcoding enables parallel quantification of Kras variant oncogenicity
Source: Nat Commun. 2017 Dec 12;8:2053. doi: 10.1038/s41467-017-01519-y (PMC5727199; doi:10.1038/s41467-017-01519-y)
Supplement: Supplementary file 1 — Supplementary Information [file 41467_2017_1519_MOESM1_ESM.pdf]

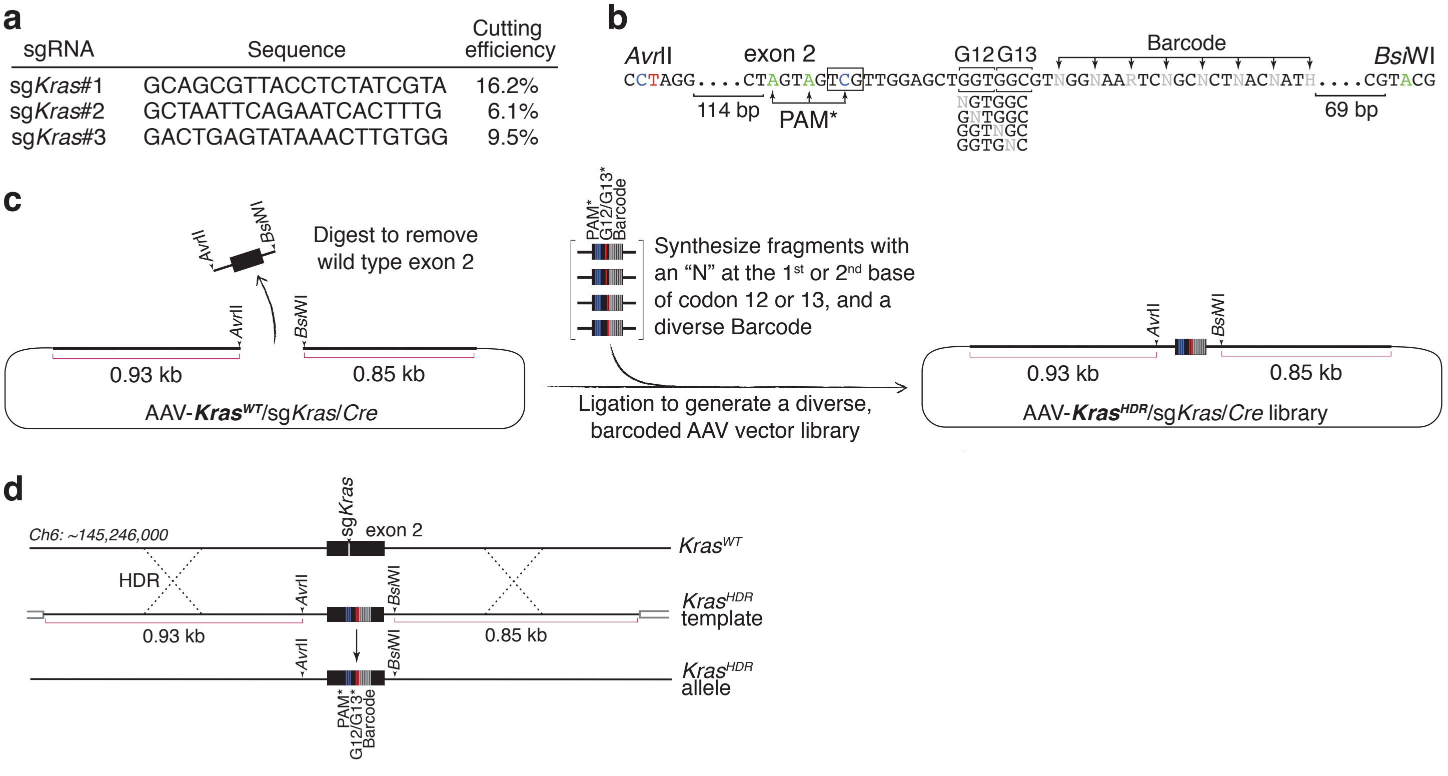

Supplementary Figure 1. Design and generation of an AAV library for multiplexed mutation of *Kras*.
a. Sequence of the three sgRNAs targeting *Kras* exon 2. Cutting efficiency of each sgRNA was determined by sequencing DNA from Cas9-expressing MEFs 48 hours after transduction with lentiviral vectors encoding each sgRNA. All three sgRNAs induced indel formation at the targeted loci. Thus, the sgRNA targeting the sequence closest to *Kras* codons 12 and 13 (sg*Kras*#3) was used for all subsequent experiments to increase the likelihood of HDR.
b. Synthesized library of dsDNA fragments containing wild type (WT) *Kras* sequence plus each of the 12 non-synonymous, single nucleotide *Kras* mutants at codons 12 and 13, silent mutations within the PAM and sgRNA homology region (PAM*), and a diverse 8-nucleotide random barcode within the wobble positions of the downstream codons for barcoding of individual tumors. Each *Kras* allele can be associated with ~2.4×10^4 unique barcodes. Fragments also contained restriction sites for cloning.
c. AAV vector library was generated by massively ligating synthesized regions into a parental AAV vector creating a barcoded pool with WT *Kras* and all 12 single-nucleotide, non-synonymous mutations in *Kras* codons 12 and 13.
d. Position of *Kras* exon 2 within the $Kras^{HDR}$ template. The lengths of the homology arms are shown.

a

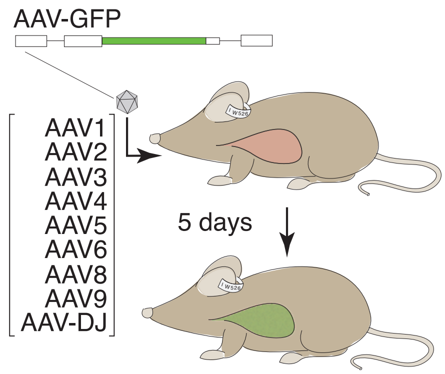

b

Capsid	Titer (vg/mL)
AAV1	$8.9x10^{10}$
AAV2	$1.8x10^{11}$
AAV3	$1.3x10^{11}$
AAV4	$1.8x10^{11}$
AAV5	$9.6x10^{10}$
AAV6	$1.2x10^{11}$
AAV8	$7.9x10^{12}$
AAV9	$4.6x10^{12}$
AAV-DJ	$7.2x10^{12}$

c

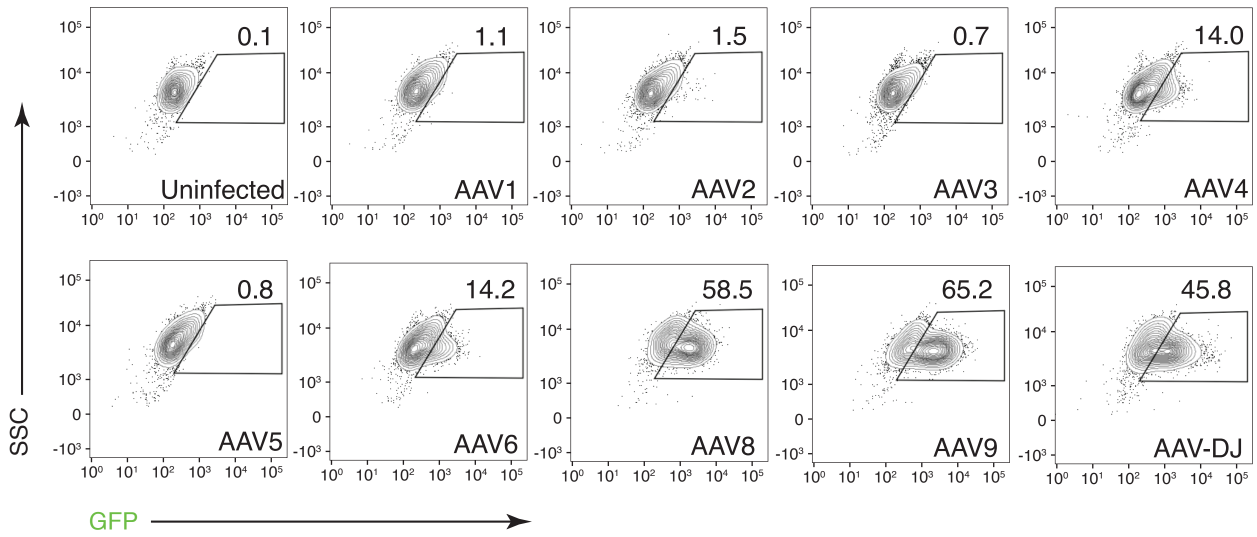

Supplementary Figure 2. Identification of an optimal AAV serotype for adult lung epithelial cell transduction.
a. Outline of the experiment to screen 9 AAV serotypes for adult lung epithelial cell transduction. An AAV vector encoding GFP was packaged with different AAV capsid serotypes and administered intratracheally to wild-type recipient mice. 5 days post-treatment, the lungs were dissociated and the percent of $GFP^{positive}$ epithelial cells was determined by flow cytometry.
b. Different AAV serotypes can be produced at different concentrations (vg = vector genomes). Our goal was to identify the AAV serotypes capable of delivering DNA templates to lung epithelial cells, which is largely dictated by both the achievable viral titer and the per virion transduction efficiency. Thus, we did not normalize the titer of the AAV serotypes before infection, but rather determined the percent infection following administrations of 60 μL of undiluted, purified virus.
c. To assess the percent of lung epithelial cells transduced by the different AAV serotypes, we dissociated lungs of transduced mice into single cell suspensions and performed flow cytometry for GFP as well as for markers of hematopoietic cells (CD45, Ter119, and F4/80), endothelial cells (CD31), and epithelial cells (EpCAM). Plots show forward scatter/side scatter (SSC)-gated, viable ($DAPI^{negative}$), lung epithelial ($CD45/Ter119/F4\text{-}80/CD31^{negative}$, $EpCAM^{positive}$) cells. The percent $GFP^{positive}$ epithelial cells in each sample is indicated above the gate. AAV8, AAV9, and AAVDJ were considerably better than all other serotypes, consistent with the high maximal titers of these serotypes. We chose to use AAV8 based on this data and the documented ability of AAV8 to efficiently transduce many other mouse cell types *in vivo*.

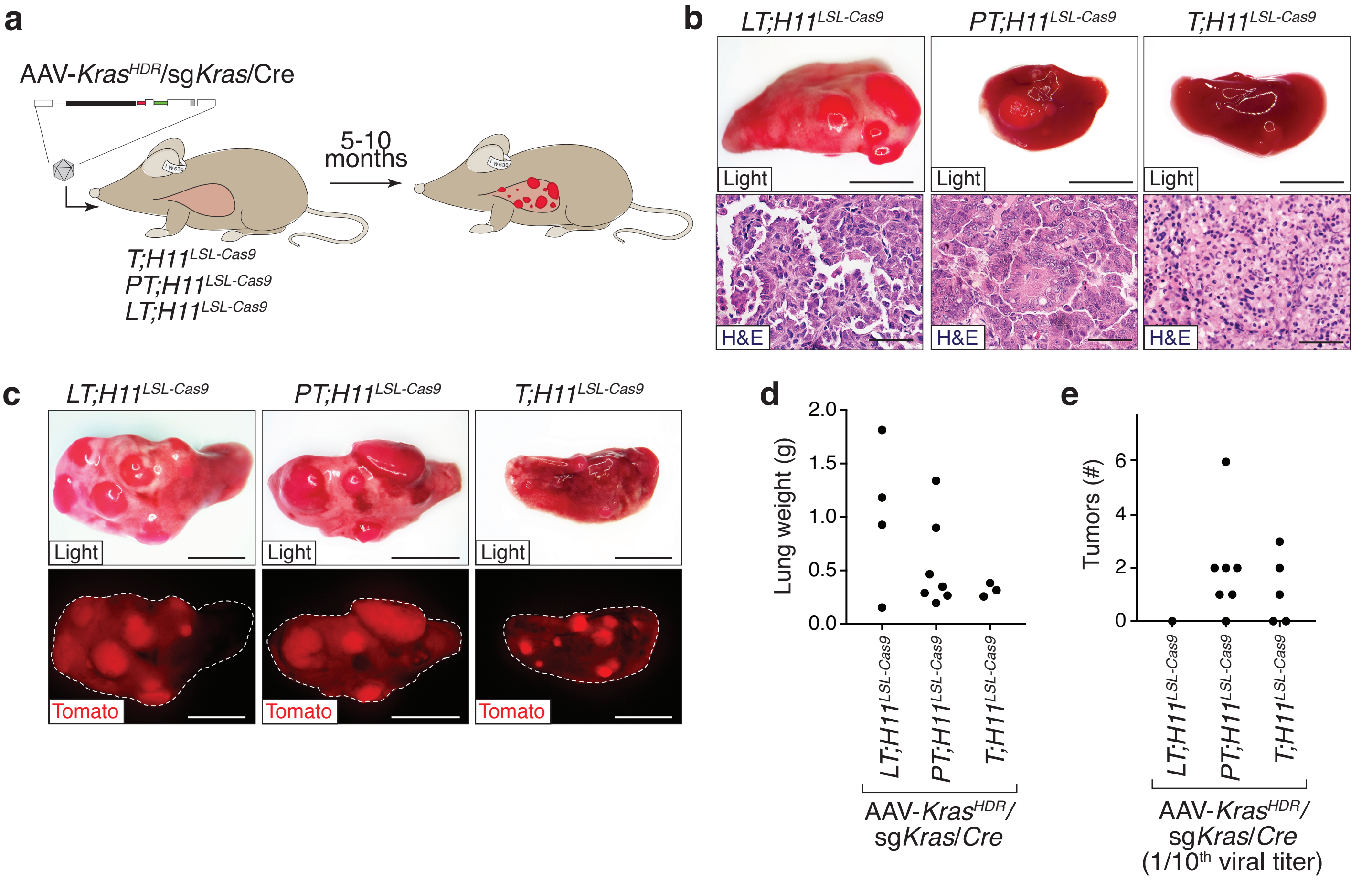

Supplementary Figure 3. AAV/Cas9-mediated HDR in somatic lung epithelial cells initiates primary tumors.

a. Schematic of the experiment to introduce point mutations into the endogenous *Kras* locus and barcode lung epithelial cells in $Lkb1^{flox/flox}$;$Rosa26^{LSL\text{-}tdTomato}$;$H11^{LSL\text{-}Cas9}$ ($LT;H11^{LSL\text{-}Cas9}$), $p53^{flox/flox}$;$Rosa26^{LSL\text{-}tdTomato}$;$H11^{LSL\text{-}Cas9}$ ($PT;H11^{LSL\text{-}Cas9}$), and $Rosa26^{LSL\text{-}tdTomato}$;$H11^{LSL\text{-}Cas9}$ ($T;H11^{LSL\text{-}Cas9}$) mice by intratracheal administration of AAV-$Kras^{HDR}$/sg*Kras*/*Cre* (8.4x10^{10} vector genomes). $LT;H11^{LSL\text{-}Cas9}$ were analyzed 5-7 months after transduction with AAV-$Kras^{HDR}$/sg*Kras*/*Cre* when they displayed signs of tumor development, while $PT;H11^{LSL\text{-}Cas9}$ mice were analyzed after 6-8 months and $T;H11^{LSL\text{-}Cas9}$ mice were analyzed at around 10 months.

b. Light images that correspond to the fluorescence images in Figure 3b. Higher magnification histology images document adenocarcinoma histology and greater nuclear atypia in the p53-deficient tumors. Upper scale bars = 5 mm. Lower scale bars = 50 μm.

c. Additional examples of AAV-$Kras^{HDR}$/sg*Kras*/*Cre*-induced lung tumors in $LT;H11^{LSL\text{-}Cas9}$, $PT;H11^{LSL\text{-}Cas9}$, and $T;H11^{LSL\text{-}Cas9}$ mice. Scale bars = 5 mm. Note that, due to the high transduction efficiency, most lung cells express Tomato, but the tumors are much brighter because of the large number and density of cells in the tumors.

d. Total lung weight in mice of each genotype with tumors initiated with AAV-$Kras^{HDR}$/sg*Kras*/*Cre*. Each dot represents a mouse.

e. Number of surface lung tumors identified under a fluorescence dissecting scope in mice of each genotype infected with AAV-$Kras^{HDR}$/sg*Kras*/*Cre* diluted 1:10. Each dot represents a mouse.

a

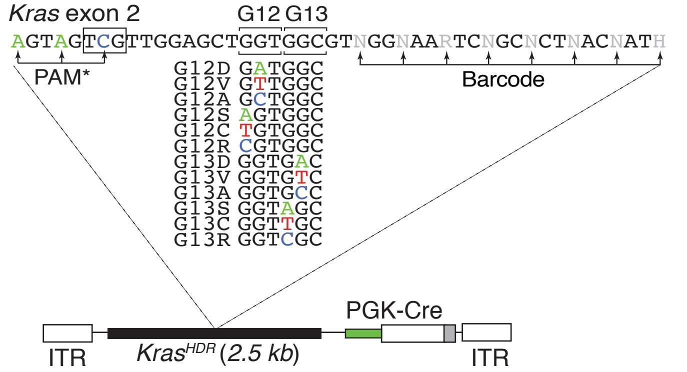

b

Kras^{HDR} allele	%
G12D	9.5
G12V	4.1
G12A	4.6
G12S	7.9
G12C	3.8
G12R	4.8
G13D	9.8
G13V	2.8
G13A	3.7
G13S	8.4
G13C	4.8
G13R	4.5
WT	31.3

c

Vector Library	Titer
AAV-*Kras*^{HDR}/sg*Kras*/*Cre*	1.4x10^{12} vg/ml
AAV-*Kras*^{HDR}/*Cre*	2.4x10^{12} vg/ml

d

Mouse genotype	Viral dilution	Mice with tumors/total mice
LT	undil.	0/5
LT	1:10	0/6
PT	undil.	1/5
PT	1:10	0/4
T	undil.	0/2
T	1:10	0/1

Supplementary Figure 4. Nuclease-free AAV-mediated HDR does not occur at a high enough rate to initiate large numbers of lung tumors.

a. Schematic of an AAV vector library containing a 2.5 kb *Kras* HDR template with the twelve single-nucleotide, non-synonymous mutations and a diverse barcode, but without the sgRNA targeting *Kras*. Since AAV vectors can stimulate homologous recombination in the absence of any targeted nucleases (Gaj, Epstein, & Schaffer, 2016), we designed this AAV vector to test whether this approach would be efficient enough to initiate tumorigenesis and to serve as a control for AAV/Cas9-mediated HDR.

b. Representation of each *Kras* codon 12 and 13 allele in the AAV-*Kras*^{HDR}/*Cre* plasmid pool. Percentages are the average of triplicate sequencing.

c. Titer of the AAV vector libraries (vg = vector genomes). Importantly, the control AAV-*Kras*^{HDR}/*Cre* viral preparation is the same or higher titer than AAV-*Kras*^{HDR}/sg*Kras*/*Cre.*

d. Quantification of the number of *Rosa26*$^{LSL\text{-}tdTomato}$ (*T*), *Lkb1*$^{flox/flox}$;*T* (*LT*), and *p53*$^{flox/flox}$;*T* (*PT*) mice that developed tumors eight months after administration of 60 μL of undiluted (undil.) or 1:10 diluted AAV-*Kras*^{HDR}/*Cre* pool.

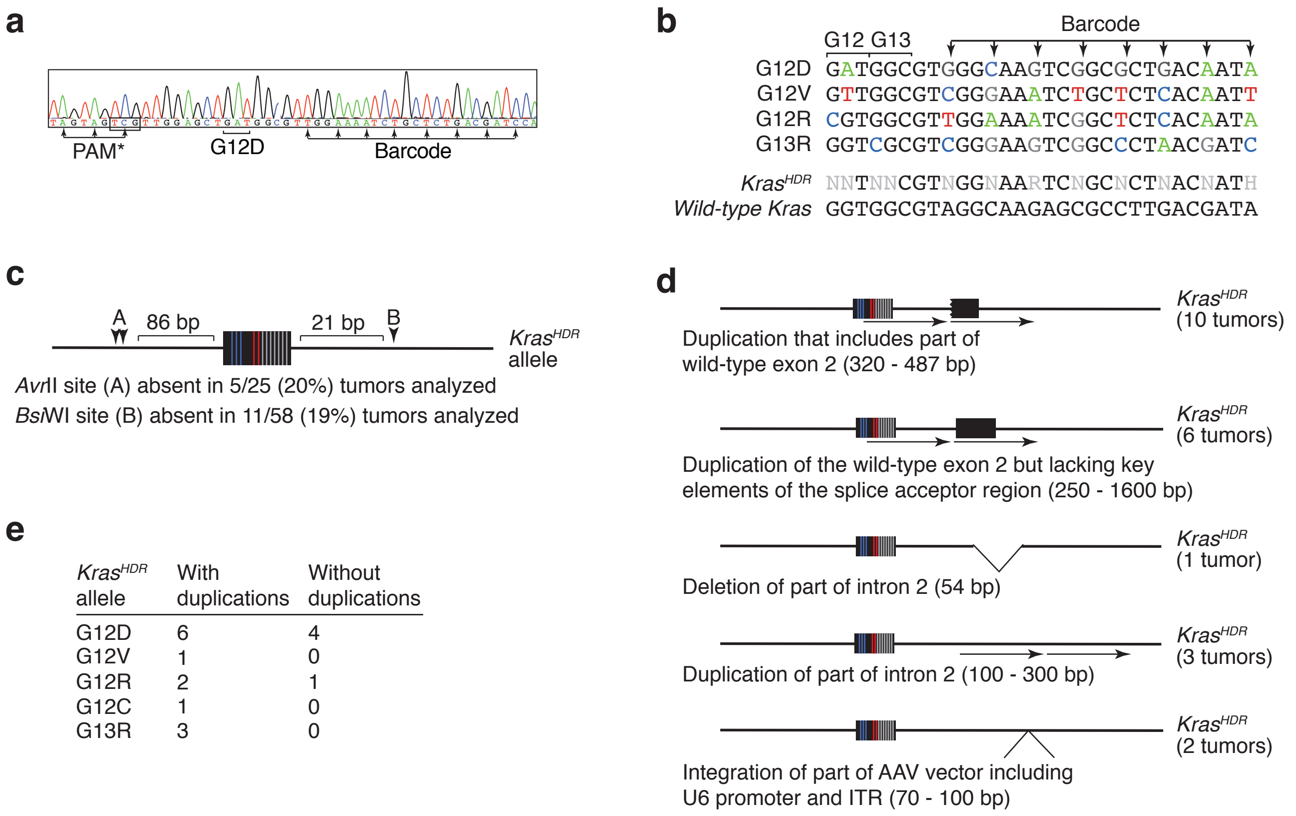

Kras^{HDR} allele	With duplications	Without duplications
G12D	6	4
G12V	1	0
G12R	2	1
G12C	1	0
G13R	3	0

Supplementary Figure 5. Analysis of individual tumors enables characterization of the oncogenic *Kras*^{HDR} alleles.
a. Example sequencing trace of a *Kras*^{HDR} allele with PAM* mutations, a G12D mutation, and a barcode.
b. Sequences of four representative oncogenic *Kras* alleles detected in individual lung tumors by Sanger sequencing. Each primary tumor analyzed had a unique variant-barcode pair, as expected given ~2.4×10^4 possible barcodes per variant. The altered bases in the AAV-*Kras*^{HDR} template sequence and the wild type *Kras* sequence are shown for reference.
c. HDR events generally occurred outside of the two engineered restriction sites. However, some tumors had *Kras* alleles consistent with recombination between exon 2 and one of the restriction sites, suggesting recombination very close to the Cas9/sg*Kras*-induced double-strand DNA break.
d. Diagram of oncogenic *Kras* alleles in individual tumors that did not undergo perfect HDR. Both perfect and imperfect HDR events were found in each mouse genotype (perfect HDR in 14/30 tumors in *LT;H11*$^{LSL\text{-}Cas9}$ mice and 3/7 tumors in *PT;H11*$^{LSL\text{-}Cas9}$ mice). Imperfect HDR events included alleles that likely integrated into the *Kras* locus through homologous recombination of the 5' end of the AAV-*Kras*^{HDR} template upstream of exon 2 and ligation of the 3' end of the AAV-*Kras*^{HDR} template to the exon 2 region immediately downstream of the Cas9/sg*Kras*-induced double-strand DNA break. This imperfect HDR resulted in insertions or deletions in the intronic sequence downstream of *Kras* exon 2. Insertions and deletions were variable in length (sizes approximated by Sanger sequencing or gel electrophoresis). The insertions sometimes included part or all of the wild type exon 2, or in rare cases, segments of the AAV-*Kras*^{HDR}/sg*Kras*/*Cre* vector. None of these partial HDR events were predicted to alter splicing from the mutant exon 2 to exon 3, consistent with the requirement for expression of the oncogenic *Kras* allele for tumor formation. Similar duplications were also observed in pancreatic tumors and sarcomas.
e. Number of lung tumors analyzed in which the indicated *Kras*^{HDR} allele contained a duplication.

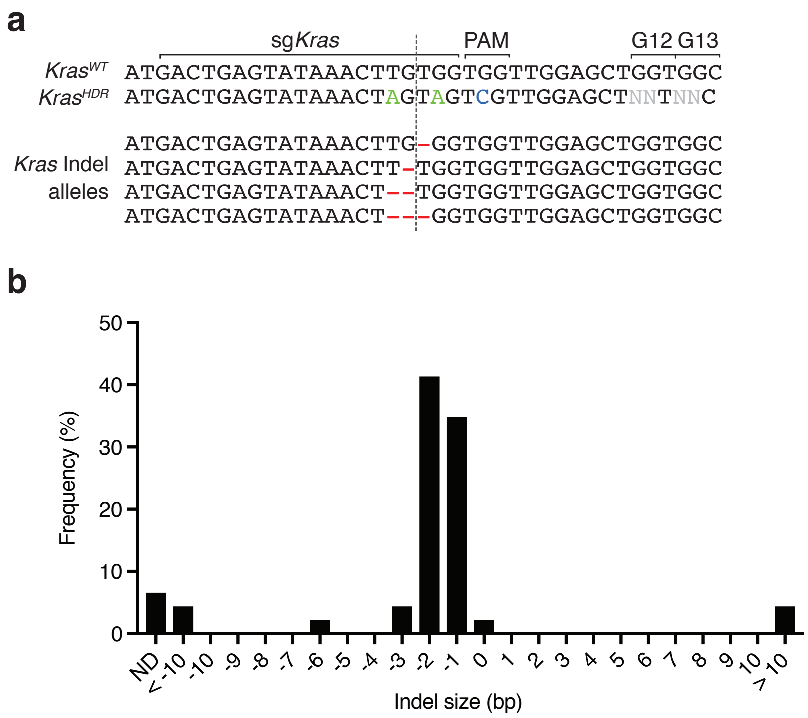

Supplementary Figure 6. Analysis of individual tumors uncovers indels in the non-HDR *Kras* allele.
a,b. The oncogenic *Kras* allele in large individual tumors from treated *PT;H11*$^{LSL\text{-}Cas9}$ and *LT;H11*$^{LSL\text{-}Cas9}$ mice was almost always accompanied by inactivation of the other *Kras* allele through Cas9-mediated indel formation in exon 2. Sanger sequencing identified indels adjacent to the PAM sequence in 45/46 (98%) individual tumors. Example indels (**a**) and a summary of all indels (**b**) are shown. The dotted line in (**a**) represents the anticipated cutting site of the sgRNA targeting *Kras*. ND indicates that a wild type allele could not be detected, which is consistent with either loss of heterozygosity, a very large insertion that precludes PCR amplification, or a large deletion that encompassed one of the primer binding sites. Small deletions or loss of heterozygosity events were also present in all pancreatic tumor masses and sarcomas analyzed.

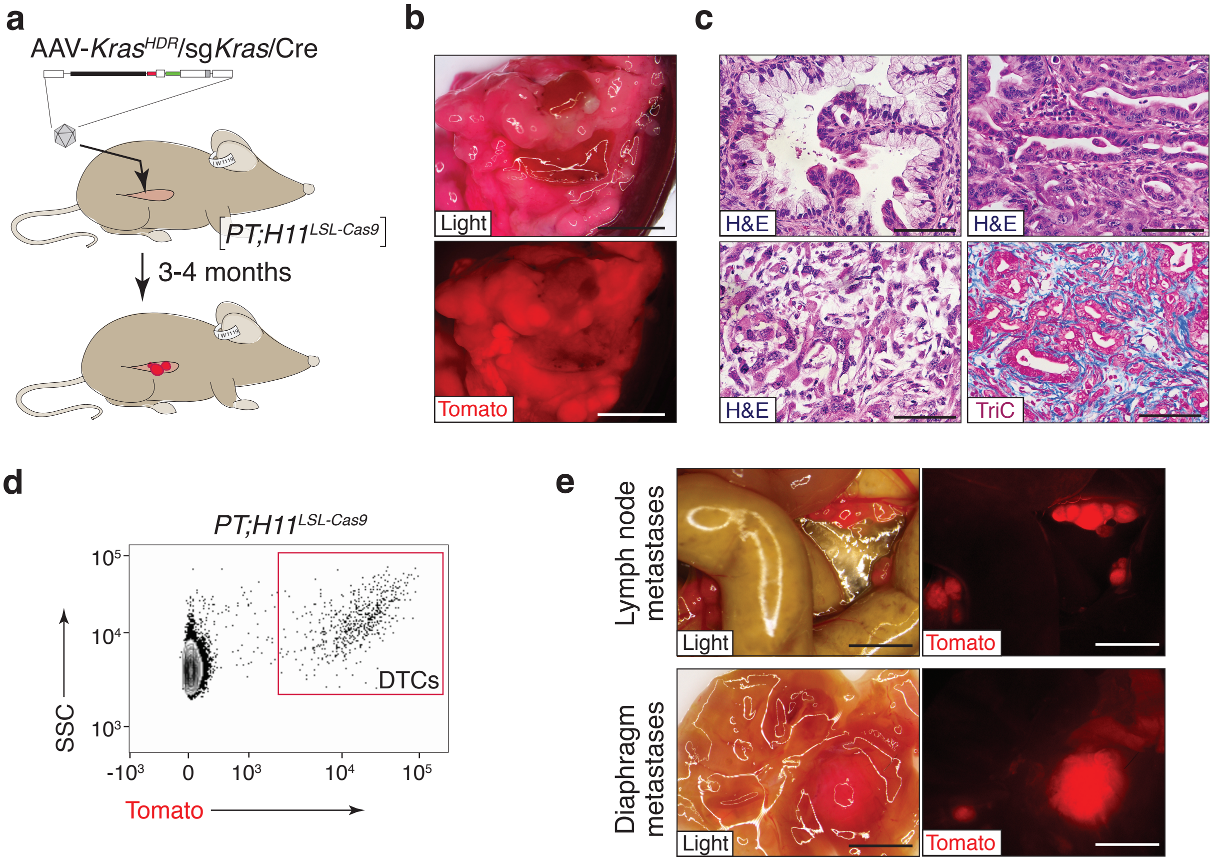

Supplementary Figure 7. HDR-mediated introduction of oncogenic mutations into the endogenous *Kras* locus in pancreatic cells leads to the formation of pancreatic ductal adenocarcinoma.

a. Schematic of retrograde pancreatic ductal injection of AAV-*Kras*^{HDR}/sg*Kras*/*Cre* (~1.7×10^{11} vector genomes) into *PT;H11*$^{LSL\text{-}Cas9}$ mice to induce pancreatic cancer.

b. Representative light and fluorescence images of pancreatic tumors that developed in *PT;H11*$^{LSL\text{-}Cas9}$ mice transduced with AAV-*Kras*^{HDR}/sg*Kras*/*Cre*. Scale bars = 5 mm.

c. Histology images of different stages of pancreatic tumor progression including a pre-cancerous PanIN lesion (upper left), a well-differentiated tumor region (top right), and poorly differentiated PDAC (bottom left). Bottom right shows the development of a collagen-rich stromal environment (stained with Trichrome) within PDAC. Scale bars = 75 μm.

d. Representative FACS plots showing Tomato^{positive} disseminated tumor cells (DTCs) in the peritoneal cavity of a *PT;H11*$^{LSL\text{-}Cas9}$ mouse with AAV-*Kras*^{HDR}/sg*Kras*/*Cre*-initiated PDAC. Plot shows forward scatter/side scatter (SSC)-gated viable cancer cells (DAPI/CD45/CD31/F4-80/Ter119^{negative}).

e. HDR-induced PDACs can progress to gain metastatic ability, seeding metastases in lymph nodes and on the diaphragm. Light and fluorescence dissecting scope images are shown. Scale bars = 3 mm.

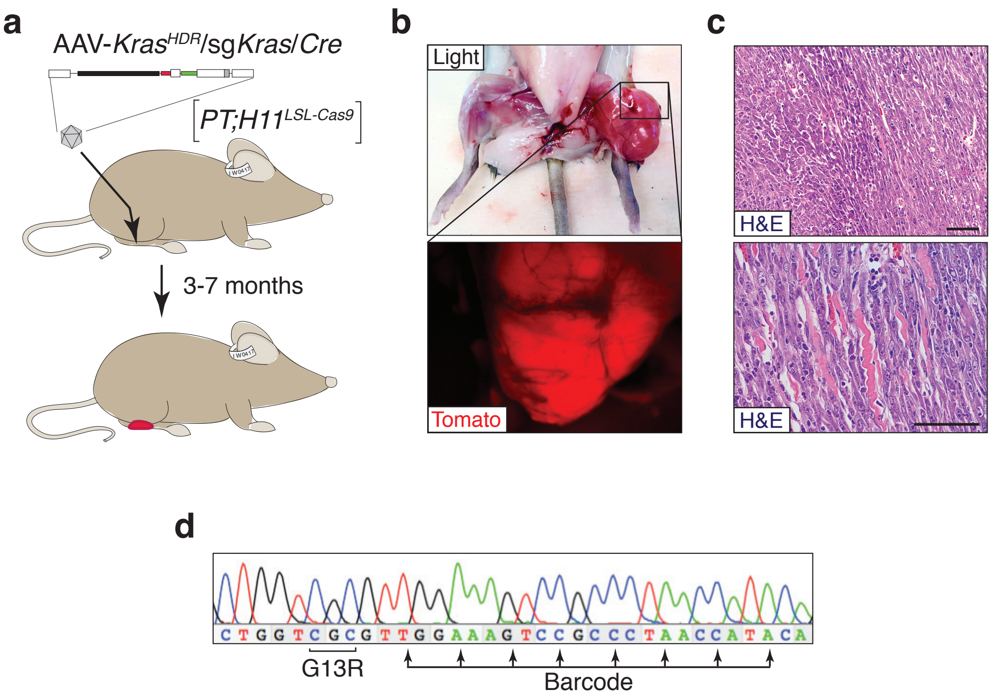

Supplementary Figure 8. HDR-mediated introduction of oncogenic mutations into the endogenous *Kras* locus in skeletal muscle induces sarcomas.

a. Schematic of intramuscular injection of AAV-*Kras*^{HDR}/sg*Kras*/*Cre* (1.6×10^{11} vector genomes) into the gastrocnemii of *PT;H11*$^{LSL\text{-}Cas9}$ mice to induce sarcomas.

b. Representative whole mount light (top panel) and fluorescence dissecting scope (bottom panel) images of mouse gastrocnemii following injection with AAV-*Kras*^{HDR}/sg*Kras*/*Cre*. Left leg has sarcoma, while the right does not, despite efficient transduction as evidenced by widespread Tomato^{positive} cells (not pictured).

c. Images of histological H&E stained sections confirming the presence of sarcoma with stereotypical histology and also invasion into the surrounding muscle. Scale bars = 75 μm.

d. Sequencing of the *Kras*^{HDR} locus in a sarcoma reveals a mutant *Kras* allele and barcode.

a

Sample	Mouse genotype	Viral dilution	Lung weight (g)	Tumor # under scope	# of tumors dissected	Bulk lung DNA in PCR (μg)	# of pooled PCR reactions
1740	*PT;H11*$^{LSL-Cas9}$	undil.	1.339	43	1	115.2	29
1740repeat	*PT;H11*$^{LSL-Cas9}$	undil.	1.339	43	1	115.2	29
2014	*PT;H11*$^{LSL-Cas9}$	undil.	0.195	5	0	16.8	5
2014repeat	*PT;H11*$^{LSL-Cas9}$	undil.	0.195	5	0	16.8	5
1734	*PT;H11*$^{LSL-Cas9}$	undil.	0.266	14	0	22.9	6
1772	*PT;H11*$^{LSL-Cas9}$	undil.	0.899	16	3	77.3	20
2091	*PT;H11*$^{LSL-Cas9}$	undil.	0.466	14	3	40.1	11
1741	*PT;H11*$^{LSL-Cas9}$	undil.	0.350	8	3	30.1	8
1778	*PT;H11*$^{LSL-Cas9}$	1:10	0.188	0	0	16.2	5
1776	*PT;H11*$^{LSL-Cas9}$	1:10	0.162	0	0	13.9	4
1767	*PT;H11*$^{LSL-Cas9}$	1:10	0.196	2	0	16.9	5
1894	*PT;H11*$^{LSL-Cas9}$	1:10	0.156	3	0	13.4	4
1894repeat	*PT;H11*$^{LSL-Cas9}$	1:10	0.156	3	0	13.4	4
2193	*LT;H11*$^{LSL-Cas9}$	undil.	ND	14	5	34.4	9
2228	*LT;H11*$^{LSL-Cas9}$	undil.	ND	7	5	16.3	5
2379	*LT;H11*$^{LSL-Cas9}$	undil.	0.927	19	6	79.7	20
2366	*LT;H11*$^{LSL-Cas9}$	undil.	1.183	36	11	101.7	26
2324	*LT;H11*$^{LSL-Cas9}$	undil.	1.814	40	10	156.0	40
2324repeat	*LT;H11*$^{LSL-Cas9}$	undil.	1.814	40	10	156.0	40
2358	*LT;H11*$^{LSL-Cas9}$	undil.	0.154	7	0	13.2	4
2080	*T;H11*$^{LSL-Cas9}$	undil.	0.316	14	0	27.2	7
2078	*T;H11*$^{LSL-Cas9}$	undil.	0.382	22	1	32.9	9
2095	*T;H11*$^{LSL-Cas9}$	undil.	0.257	21	0	22.1	6

b

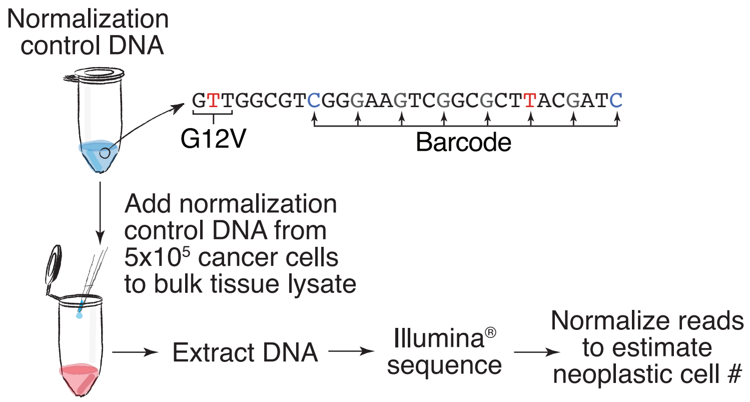

Supplementary Figure 9. Samples and preparation for high-throughput sequencing of bulk lung tissue to quantify the size and number of lung tumors with each mutant *Kras* allele.

a. Bulk lung tissue samples from mice with AAV-*Kras*^{HDR}/sg*Kras*/*Cre*-initiated tumors for Illumina® sequencing of barcoded *Kras*^{HDR} alleles. Sample name, mouse genotype, and dilution of AAV-*Kras*^{HDR}/sg*Kras*/*Cre* are indicated. The weight, tumor number, number of dissected tumors, as well as the amount of DNA amplified and the number of PCR reactions pooled for Illumina® sequencing for each sample are shown. Repeat samples are technical replicates. ND = No data.

b. Pipeline to estimate the number of neoplastic cells in individual tumors directly from bulk lung samples. An aliquot of DNA extracted from $5x10^5$ cells possessing a known barcode is added to each bulk lung sample after tissue homogenization. This DNA serves as a normalization control: sequencing read counts from each uniquely barcoded lung tumor can be converted into an estimate of cell number by comparison to the sequencing read counts from the normalization control DNA, which is known to correspond to $5x10^5$ cells.

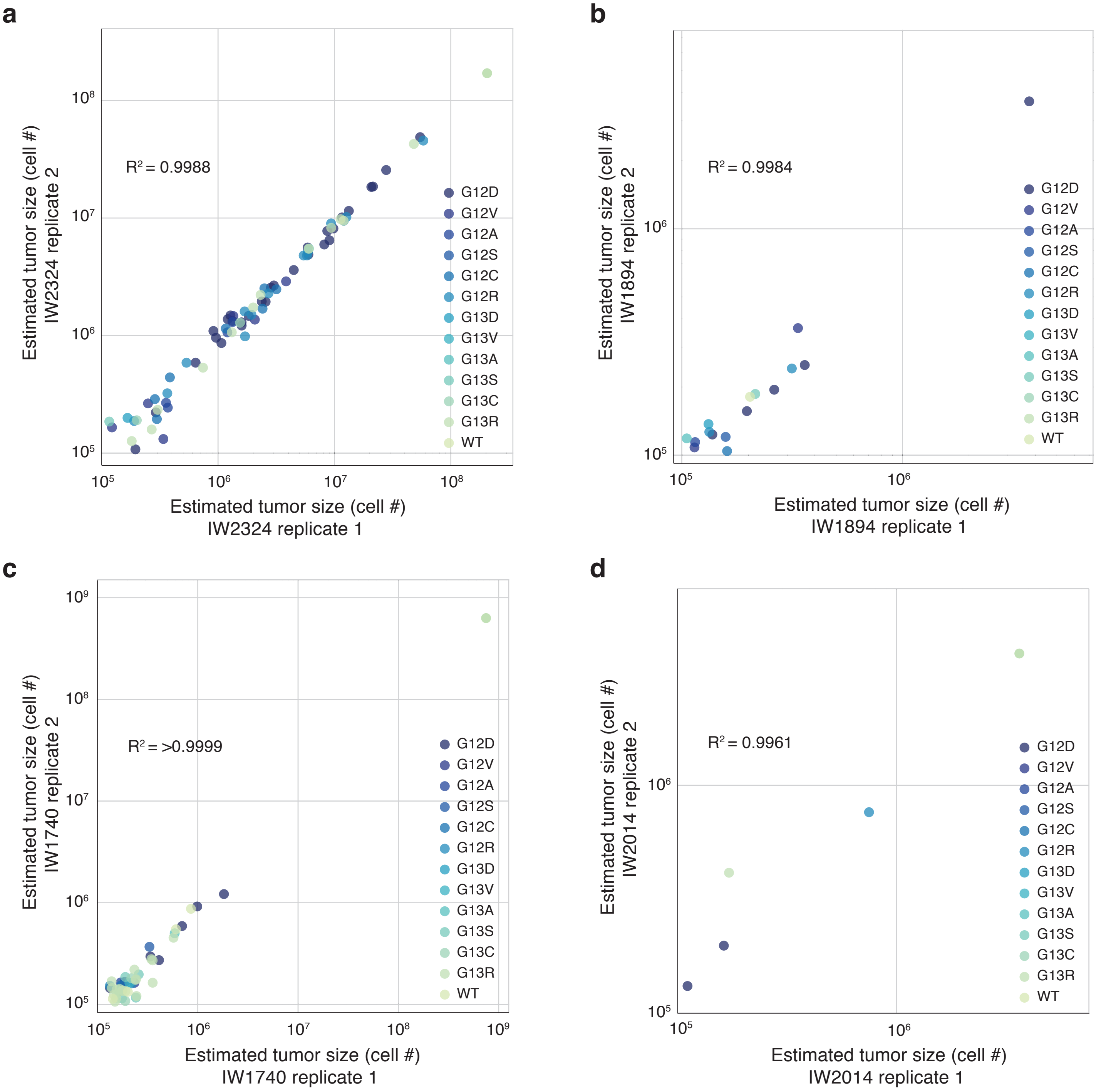

Supplementary Figure 10. Reproducibility of barcode sequencing-based parallel analysis of tumor genotype, size, and number from bulk tissue.
a-d. Regression plot of individual tumors with the indicated *Kras*^{HDR} allele and a unique barcode detected by high-throughput sequencing across technical replicates (i.e. independent DNA extraction from bulk tissue lysate and PCR reactions). Each dot represents a tumor. Replicates in **a** and **b** were PCR amplified using primers with different multiplexing tags, but were run on the same sequencing lane. Replicates in **c** and **d** were PCR amplified using the same primers, but were run on different sequencing lanes. Mice with above average tumor burden (**a**,**c**) and below average tumor burden (**b**,**d**), as estimated by bulk lung weight, were analyzed to confirm the technical and computational reproducibility of this pipeline across samples of variable tumor number.

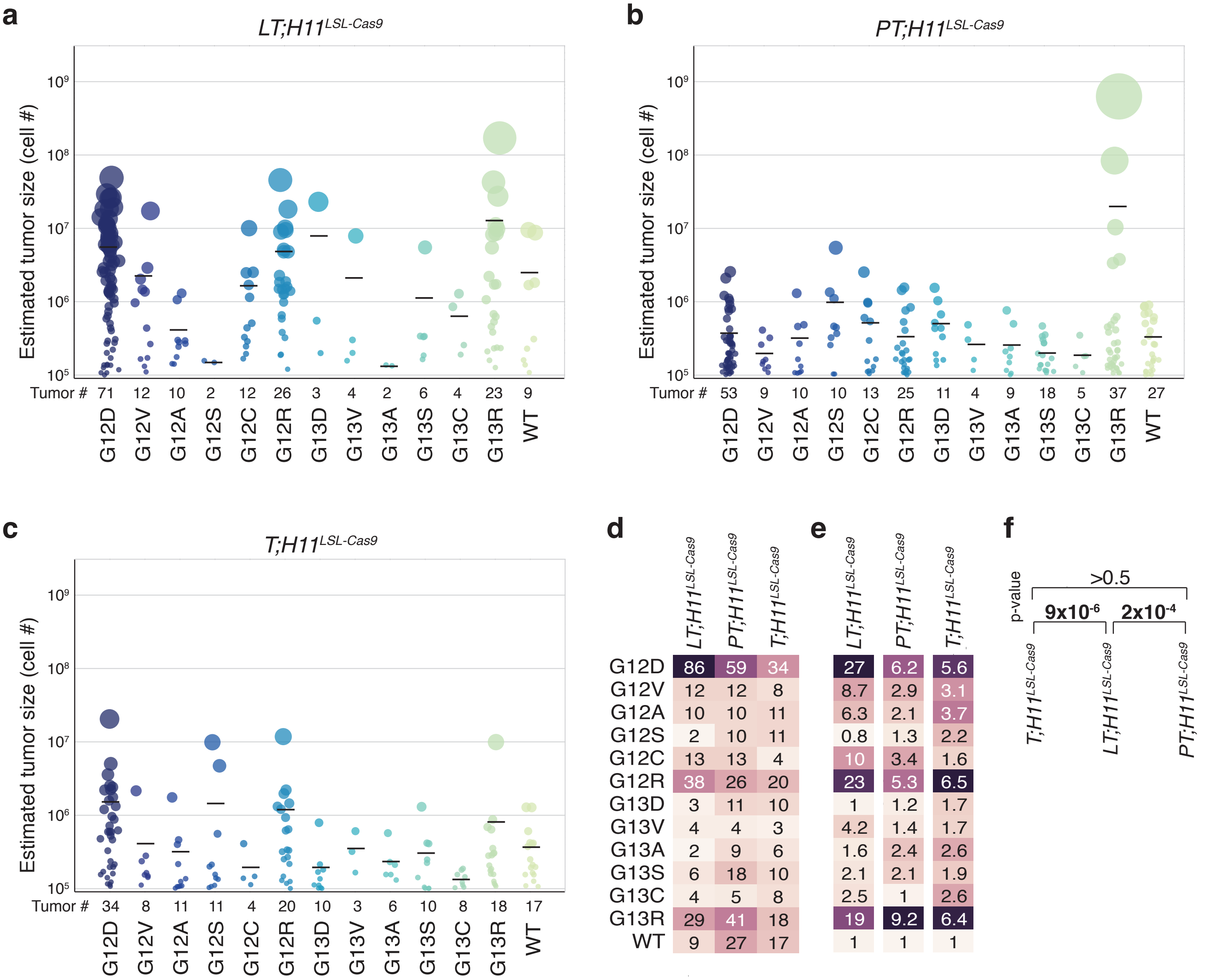

d

	*LT;H11*LSL-Cas9	*PT;H11*LSL-Cas9	*T;H11*LSL-Cas9
G12D	86	59	34
G12V	12	12	8
G12A	10	10	11
G12S	2	10	11
G12C	13	13	4
G12R	38	26	20
G13D	3	11	10
G13V	4	4	3
G13A	2	9	6
G13S	6	18	10
G13C	4	5	8
G13R	29	41	18
WT	9	27	17

e

	*LT;H11*LSL-Cas9	*PT;H11*LSL-Cas9	*T;H11*LSL-Cas9
G12D	27	6.2	5.6
G12V	8.7	2.9	3.1
G12A	6.3	2.1	3.7
G12S	0.8	1.3	2.2
G12C	10	3.4	1.6
G12R	23	5.3	6.5
G13D	1	1.2	1.7
G13V	4.2	1.4	1.7
G13A	1.6	2.4	2.6
G13S	2.1	2.1	1.9
G13C	2.5	1	2.6
G13R	19	9.2	6.4
WT	1	1	1

Supplementary Figure 11. High-throughput barcode sequencing of tumors from bulk lung tissue uncovers diverse numbers and sizes of tumors with different *Kras* alleles.

a-c. Tumor size distributions of *Kras* variants in *LT;H11*$^{LSL\text{-}Cas9}$ (N=6) (**a**), *PT;H11*$^{LSL\text{-}Cas9}$ (N=6) (**b**), and *T;H11*$^{LSL\text{-}Cas9}$ (N=3) (**c**) mice transduced with AAV-*Kras*^{HDR}/sg*Kras*/*Cre*. Each dot represents a tumor with a unique *Kras* variant-barcode pair. The size of each dot is proportional to the size of the tumor it represents, which is estimated by normalizing tumor read counts to the normalization control reads counts. Black bars representing the mean size of tumors harboring each *Kras*^{HDR} allele are shown for reference. By design, WT *Kras*^{HDR} alleles were approximately four times more prevalent than average in AAV-*Kras*^{HDR}/sg*Kras*/*Cre* library. This results in a visual overrepresentation of lesions with WT *Kras*^{HDR} alleles, of which some are thought to be hitchhikers in tumors with oncogenic *Kras*^{HDR} alleles (see Methods).

d,e. Tables of raw (**d**) and normalized (**e**) number of tumors harboring each *Kras* variant across each genotype. Note that the values in (**d**) are a sum of the number of tumors identified by high-throughput sequencing of bulk lung tissue as well as those identified by individual tumor dissection and analysis. In (**e**), the number of tumors harboring each *Kras* variant is normalized to the initial representation of each variant in the AAV plasmid library and to the number of lesions harboring a WT allele within the same genotype of mice. Note that the color intensity scale of the heatmaps in (**e**) is unique to each mouse genotype.

f. P-values from a two-sided multinomial Chi-squared test of the number of lung tumors with each *Kras* variant across different genotypes, indicating that there are significant differences between the spectrum of *Kras* mutations observed in *LT;H11*$^{LSL\text{-}Cas9}$ mice compared to the other genotypes of mice. Significant p-values ($p < 0.05$) are bold.

a

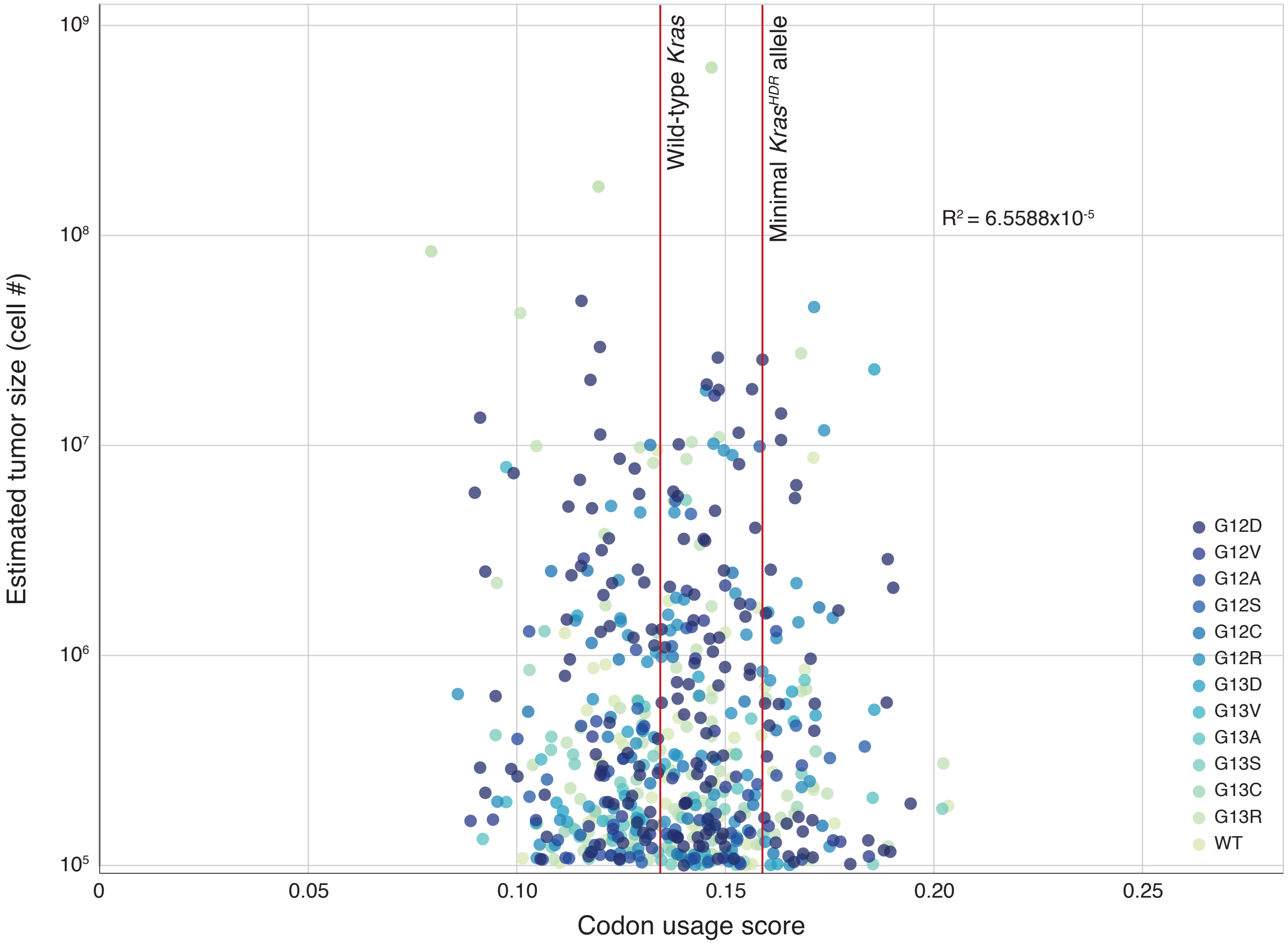

Supplementary Figure 12. Lung tumor size does not correlate with codon usage in the *Kras*^{HDR} barcode region. **a.** Broad changes in codon usage can impact Kras-driven tumorigenesis (Lampson *et al*., 2013; Pershing *et al*., 2015). The barcode region of each *Kras*^{HDR} allele contains three anchor bases that are different from wild-type *Kras* as well as random nucleotides in the eight positions that make up the barcode. This plot depicts the size of individual tumors with each *Kras*^{HDR} allele (see Supplementary Fig. 11a-c) plotted against the "codon usage score" of the unique barcode region in each tumor. The codon usage score is the sum of the mouse codon frequencies across the 22 bp barcode region in the *Kras*^{HDR} allele in each tumor (see Methods) (Nakamura, Gojobori, & Ikemura, 2000). Vertical red lines represent the codon usage score for wild-type *Kras* and for a *Kras*^{HDR} allele containing the three universal anchor bases, but the wild-type bases at all eight positions of the barcode (minimal *Kras*^{HDR} allele).

a

Sample	Mouse genotype	Viral dilution	Tissue
2210T1	$PT;H11^{LSL\text{-}Cas9}$	undil.	Pancreas region
2210T2	$PT;H11^{LSL\text{-}Cas9}$	undil.	Pancreas region
2210T3	$PT;H11^{LSL\text{-}Cas9}$	undil.	Pancreas region
2210LNM	$PT;H11^{LSL\text{-}Cas9}$	undil.	Lymph nodes
2013T	$PT;H11^{LSL\text{-}Cas9}$	undil.	Pancreas region
2013DM1	$PT;H11^{LSL\text{-}Cas9}$	undil.	Diaphragm met
2013DM2	$PT;H11^{LSL\text{-}Cas9}$	undil.	Diaphragm met

b

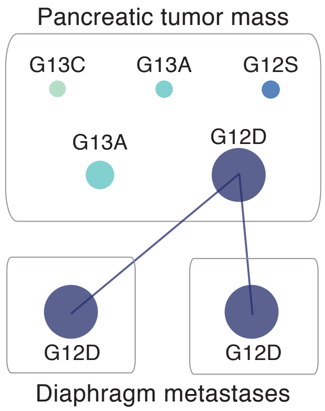

Supplementary Figure 13. High-throughput sequencing of pancreatic tumor masses and metastases identifies oncogenic *Kras* mutants.

a. Bulk pancreas tissue and metastasis samples from mice with pancreatic tumors initiated by AAV-$Kras^{HDR}$/sg*Kras*/*Cre* by retrograde pancreatic ductal injection for Illumina sequencing of barcoded $Kras^{HDR}$ alleles. Sample name, mouse genotype, viral dilution, and tissue are indicated. The $Kras^{HDR}$ alleles present in the primary tumor masses as well as metastases were analyzed by Illumina® sequencing after FACS isolating FSC/SSC-gated viable cancer cells (DAPI/CD45/CD31/F4-80/$Ter119^{negative}$) from these samples.

b. High-throughput sequencing of the primary pancreatic tumor mass and metastases from a $PT;H11^{LSL\text{-}Cas9}$ mouse with AAV-$Kras^{HDR}$/sg*Kras*/*Cre*-initiated PDAC. This uncovered diverse mutant *Kras* alleles and enabled the establishment of clonal relationships between primary tumors and their metastatic descendants. Each dot represents a tumor with the indicated *Kras* variant and a unique barcode within the indicated sample. Dots that are linked by a colored line harbor the same barcode, suggesting that they are clonally related. The size of each dot is scaled according to the size of the tumor that it represents (diameter of the dot = relative $size^{1/4}$). Since the size of pancreatic tumors is not normalized to a control, tumors sizes can only be compared within the same sample. Thus, the largest tumor in each sample is set to the same standard size.

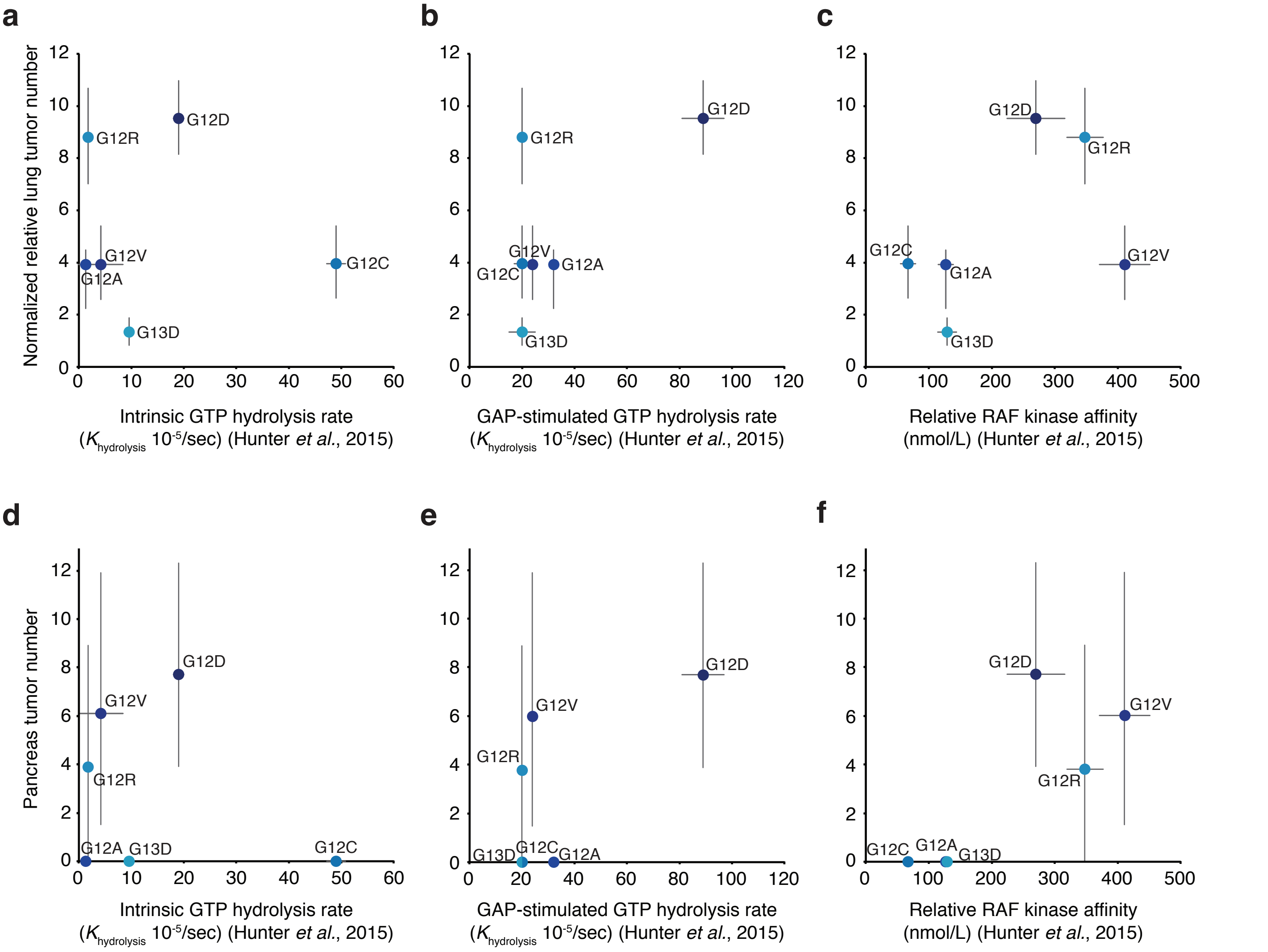

Supplementary Figure 14. Relationship between the *in vivo* oncogenicities and biochemical behaviors of Kras mutants.

a-c. Number of lung tumors in mice transduced with AAV-*Kras*^{HDR}/sg*Kras*/*Cre* as a function of the indicated biochemical property reported in Hunter *et al*., 2015. Lung tumor number is normalized to the initial representation of each *Kras* variant in the AAV-*Kras*^{HDR}/sg*Kras*/*Cre* plasmid pool and relative to WT (see Figure 6b). Vertical bars represent the 95% confidence interval for the normalized relative lung tumor number. Horizontal bars represent the standard error of the mean of three replicate experiments as described in Hunter *et al*., 2015. P120GAP was used to determine GAP-stimulated GTP hydrolysis rates (Hunter *et al*., 2015).

d-f. Number of pancreatic tumors in mice transduced with AAV-*Kras*^{HDR}/sg*Kras*/*Cre* as a function of the indicated biochemical property reported in Hunter *et al*., 2015. Pancreatic tumor number is normalized to the initial representation of each *Kras* variant in the AAV-*Kras*^{HDR}/sg*Kras*/*Cre* plasmid pool (see Figure 7b). Vertical bars represent the 95% confidence interval for normalized pancreas tumor number. Horizontal bars represent the standard error of the mean of three replicate experiments as described in Hunter *et al*., 2015. P120GAP was used to determine GAP-stimulated GTP hydrolysis rates (Hunter *et al*., 2015).

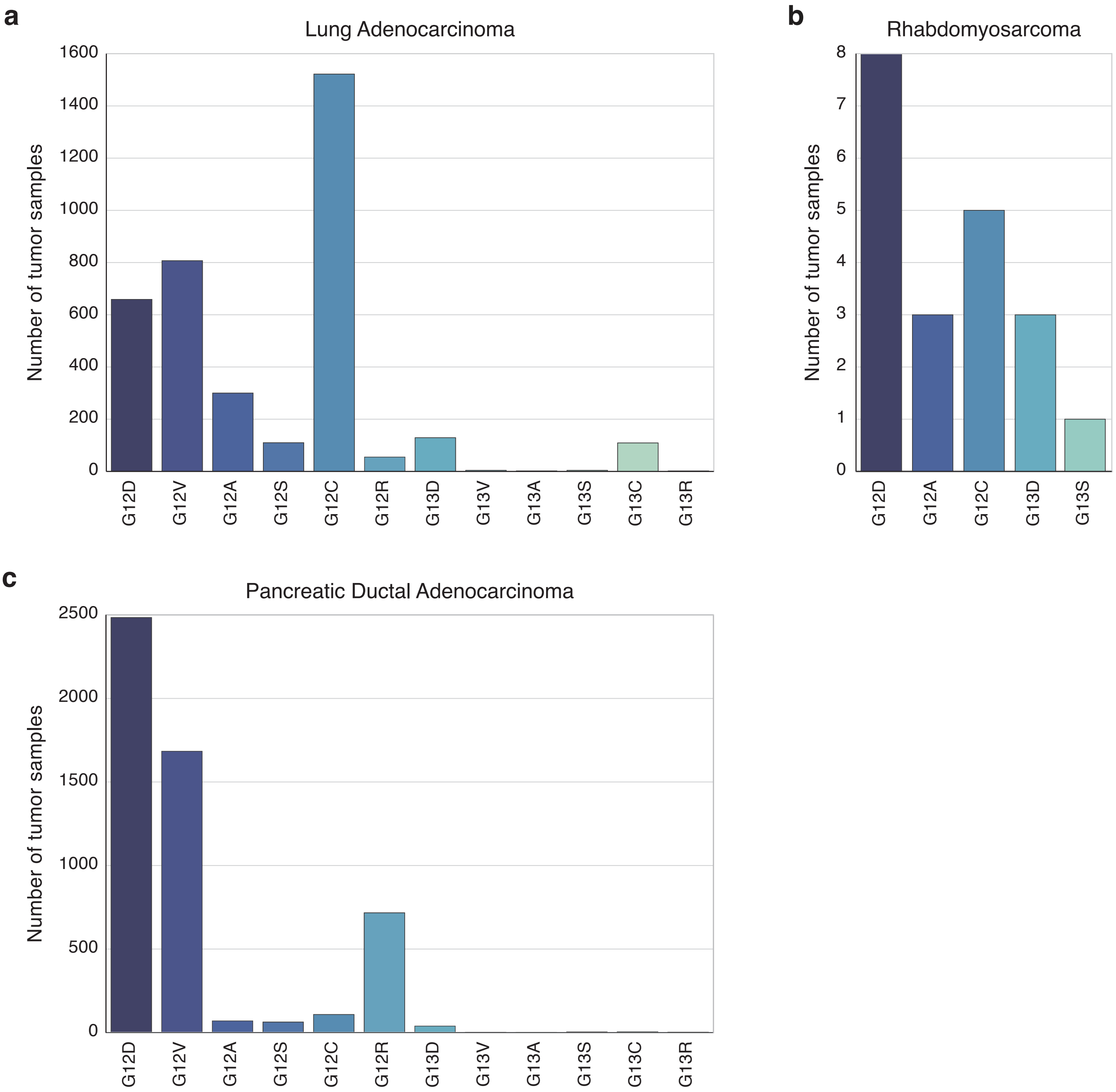

Supplementary Figure 15. Prevalence of *KRAS* mutations in human lung adenocarcinoma, pancreatic ductal adenocarcinoma, and rhabdomyosarcoma.
a-c. Prevalence of *KRAS* mutations in the indicated cancer types extracted from pooled data from the Catalogue Of Somatic Mutations In Cancer (COSMIC), AACR Project Genomics Evidence Neoplasia Information Exchange (GENIE) databases, and additional individual studies (see Supplementary Data 4 and Methods).

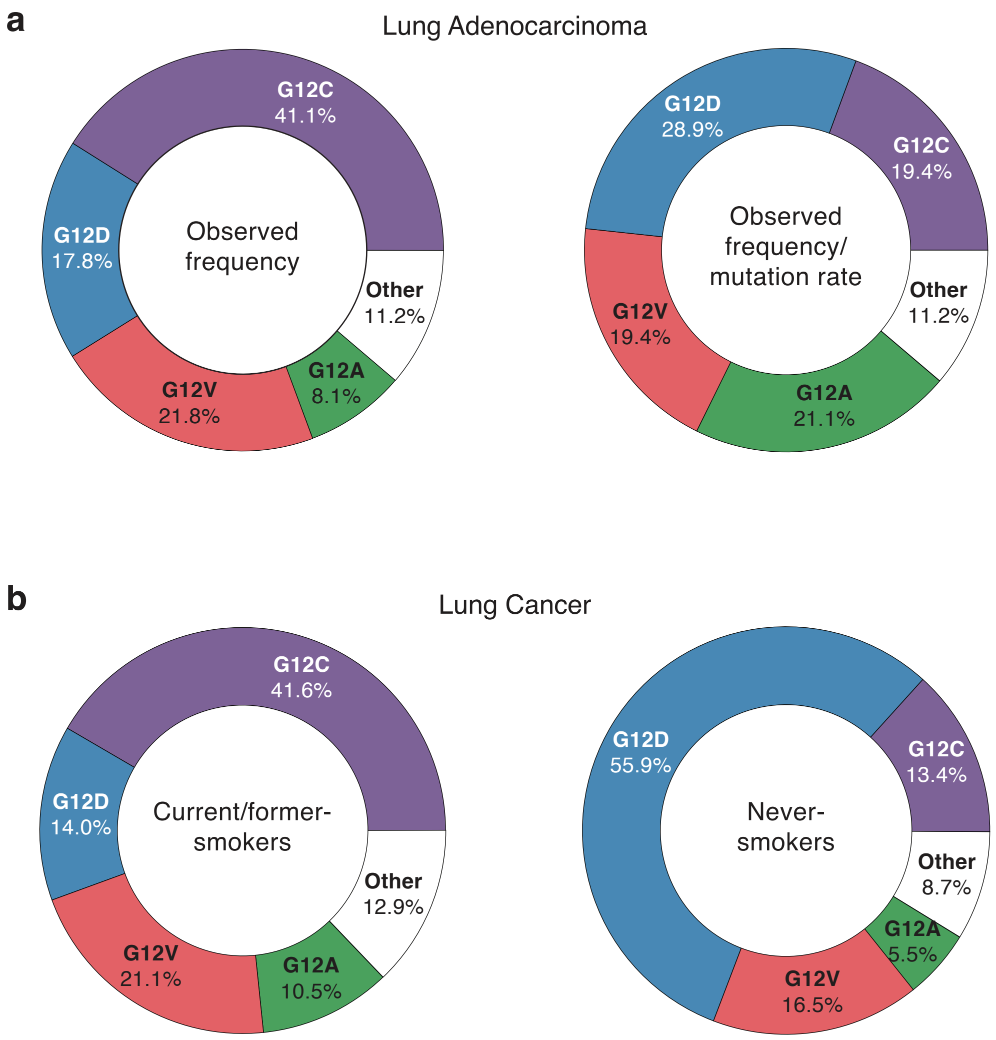

Supplementary Figure 16. The spectrum of KRAS mutations in human lung cancer is influenced by mutational processes.
a. Prevalence of KRAS amino acid 12 and 13 substitutions in lung adenocarcinomas. The observed frequency of each mutation in human lung adenocarcinoma is shown in the left pie chart. The right pie chart represents the observed frequency normalized to the estimated relative probability of each *KRAS* mutation occuring in the cell of origin of lung adenocarcinoma. The relative probabilities are estimated from the nucleotide substitution rates of tumor-extrinsic mutational processes affecting the lung cancer genome as reported in Campbell *et al.*, 2016 (see Supplementary Data 5 and Methods).
b. Prevalence of KRAS amino acid 12 and 13 substitutions in lung adenocarcinomas by smoking status (see Supplementary Data 6).

a

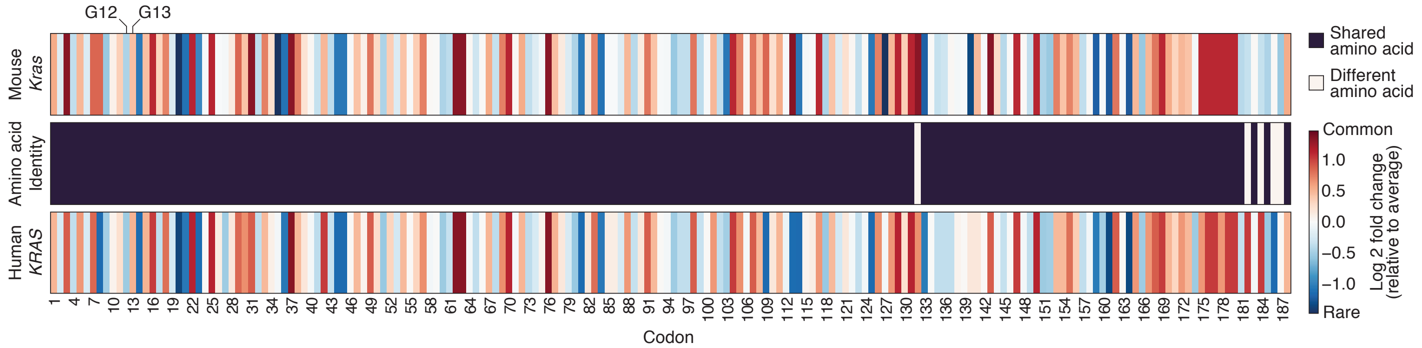

Supplementary Figure 17. Comparison of codon and amino acid usage in mouse Kras and human KRAS.
a. The amino acid identity of mouse Kras (GRCm38.p4 CCDS20693.1; isoform 4B) versus human KRAS (GRCh38.p7 CCDS8702.1; isoform 4B) is shown in the middle row. Purple bars represent shared amino acids while white bars represent divergent amino acids. Relative codon usage of mouse Kras (top row) and human KRAS (bottom row) is shown. White indicates codons that are used at an unbiased frequency (i.e., 1/64), while shades of red indicate relatively increased codon usage and blue shades indicate relatively decreased codon usage (in mice for the top row and in humans for the bottom row). Codons corresponding to KRAS amino acids G12 and G13 are indicated.
