# Supplementary material for: Multiplexed in vivo homology-directed repair and tumor barcoding enables parallel quantification of Kras variant oncogenicity
Source: Nat Commun. 2017 Dec 12;8:2053. doi: 10.1038/s41467-017-01519-y (PMC5727199; doi:10.1038/s41467-017-01519-y)
Supplement: Supplementary file 2 — Description of Additional Supplementary Files [file 41467_2017_1519_MOESM2_ESM.pdf]

Description of Supplementary Files

File Name: Supplementary Data 1
Description: **Data from Illumina® sequencing of barcoded tumors in bulk lung tissue (corresponding to Supplementary Fig. 11a-c).** The Kras variant and barcode identified in tumors from *LT;H11*$^{LSL-Cas9}$ (N=6), *PT;H11*$^{LSL-Cas9}$ (N=6), and *T;H11*$^{LSL-Cas9}$ (N=3) mice transduced with undiluted AAV-*Kras*^{HDR}*/sgKras/Cre* virus are shown (see Supplementary Fig. 9a for sample info). Tumor size (i.e. neoplastic cell number) was estimated using a normalization control, normalized to the representation of each variant in the plasmid library, and corrected for sequencing errors and barcode collisions (see Methods). A cell number cutoff of 100000 was used to call tumors (see Methods).

File Name: Supplementary Data 2
Description: **Normalized relative lung tumor number (corresponding to Fig. 6b-e).** Number of lung tumors harboring each mutant *Kras* allele normalized to its initial representation (mutant representation in the AAV plasmid library divided by WT representation in the AAV plasmid library) and relative to WT (mutant tumor # divided by WT tumor #) are listed ("Normalized relative lung tumor #"). Bootstrapped 95% confidence intervals are also listed. p-values represent a two-sided Fisher's exact test for enrichment relative to WT or to G12D. Normalized relative lung tumor numbers were generated from pooled data from all mouse genotypes (N=15), or individually from *LT;H11*$^{LSL-Cas9}$ (N=6), *PT;H11*$^{LSL-Cas9}$ (N=6), or *T;H11*$^{LSL-Cas9}$ (N=3) mice.

File Name: Supplementary Data 3
Description: **Data from Illumina® sequencing of barcoded tumors in bulk pancreas and lymph node tissue.** The Kras variant and barcode identified in primary tumors and metastases from *PT;H11*$^{LSL-Cas9}$ (N=2) mice transduced with undiluted AAV*Kras*^{HDR}*/sgKras/Cre* virus are shown. The raw number of Illumina® read counts that mapped to each unique variant-barcode pair is shown ("Observed read counts"). Since a normalization control was not included in these samples, only relative tumor sizes within each sample can be compared ("Relative read counts"; see Methods). A read count cutoff of two times the number of reads from the most abundant WT *Kras*^{HDR} allele was used to call tumors (see Methods).

File Name: Supplementary Data 4
Description: **Prevalence of *KRAS* mutations in human lung adenocarcinoma, pancreatic ductal adenocarcinoma, and rhabdomyosarcoma (corresponding to Supplementary Fig. 15).** Prevalence of *KRAS* mutations in the indicated cancer types from the Catalogue Of Somatic Mutations In Cancer (COSMIC), AACR Project Genomics Evidence Neoplasia Information Exchange (GENIE), and additional individual studies (see Methods).

File Name: Supplementary Data 5
Description: **Probability of specific *KRAS* mutations resulting from tumor extrinsic mutational processes.** The relative probabilities of *KRAS* codon 12 and 13 mutations occurring in the cell of origin of lung adenocarcinoma estimated from the nucleotide substitution rates of tumor-extrinsic mutational processes affecting the lung cancer genome, as reported in Campbell *et al.*, 2016 (see Methods).

File Name: Supplementary Data 6
Description: **Prevalence of *KRAS* codon 12 and 13 mutations in lung cancer in current/former-smokers versus never-smokers (corresponding to Supplementary Fig. 16b).** *Note: since raw *KRAS* mutation counts were not provided in Yu *et al.* 2015, mutation counts were estimated from the reported *KRAS* codon 12 and 13 mutation frequencies and the number of patients sampled.
